# Faunistic Composition, Ecological Properties and Zoogeographical Composition of the Family Elateridae (Coleoptera) of the Central Anatolian Region of Turkey

**DOI:** 10.1673/031.011.5701

**Published:** 2011-05-04

**Authors:** Mahmut Kabalak, Osman Sert

**Affiliations:** Hacettepe University Faculty of Science, Department of Biology, 06532 Beytepe Ankara, Turkey

**Keywords:** Click beetles, species composition, phenology, abundance, habitat preference, distribution

## Abstract

The focus of this study was to understand the faunistic composition, ecological properties and zoogeographical composition of Elateridae (Coleoptera) of the Central Anatolian region. 72 species belonging to seven subfamilies and 25 genera were identified. The major part of the Elateridae fauna of the Central Anatolian region is formed by the subfamilies Elaterinae and Cardiophorinae. The genus *Cardiophorus* was the most species-rich genus. The species composition of the Elateridae fauna of the Central Anatolian region is partially consistent with known Elateridae fauna of Turkey. The Central Anatolian region shares most species with the European part of the Western Palaearctic as does the Elateridae fauna of Turkey. Detailed localities of nine species are given for the first time for Turkey, with emphasis on the Central Anatolian region.

## Introduction

The family Elateridae is the ninth biggest family of Coleoptera and belongs to the superfamily Elateroidea (Lawrence, 1982). According to various authors (Lawrence, 1982; Lodos, 1998; Demirsoy, 1999; Booth et al., 1990; Laibner, 2000), the family Elateridae has 6,000–10,000 species. In Turkey, there are eight subfamilies, 65 genera and more than 450 species belonging to the Elateridae (Mertlik and Platia, 2008; Kabalak and Sert, 2009; Platia and Gudenzi, 2009; Platia et al., 2009; Schimmel et al., 2009). The number of species of Elateridae in Turkey is increasing rapidly.

**Figure 1.**
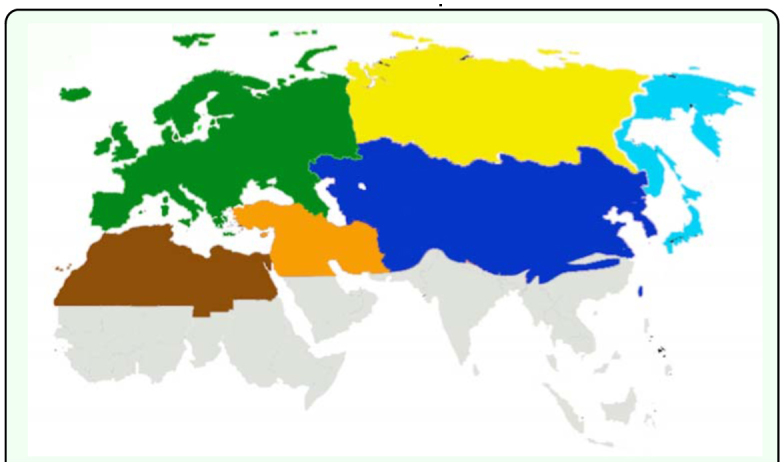
Map of Palaearctic Region (Green: European part of Western Palaearctic, Brown: North Africa, Yellow: Siberia, Navy Blue: Middle Asia, Orange: Middle East, Light Blue: Far East) (modified by authors) (http://upload.wikimedia.org/wikipedia/commons/thumb/f/fb/Ecozone-Biocountries-Palearctic.svg/800px-Ecozone-Biocountries-Palearctic.svg.png). High quality figures are available online.

Turkey is at the intersection of three continents (Asia, Africa, and Europe) (Figure 1) and it was divided into seven geographic regions (three inner regions and four coastal regions) at the first geography congress of Turkey in Ankara in 1941. Geographic regions were determined according to several criteria, including the presence of sea around three sides of Turkey; parallel extension of high mountains to coasts; separation of the middle part of Anatolia from the sea by high mountains thus climatic and floristic differentiations occur between inner parts and coastal parts; and the distribution of agriculture types and transportation systems and house types. The first four of the seven regions determined to be adjacent to the sea are the Black Sea region, Marmara region, Aegean region and Mediterranean region. The other three regions are named according to their location in the whole of Anatolia (Central Anatolian region, Eastern Anatolian region and Southeastern Anatolian region) (www.cografya.gen.tr/egitim/bolgeler/).

**Figure 2.**
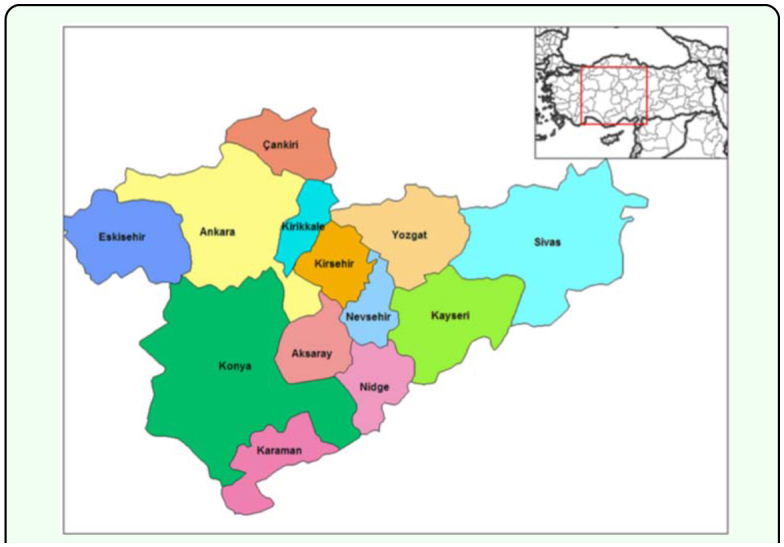
Map of research area (Provinces of Central Anatolian region are shown by using different colours). (http://www.sosyalokulu.com/konuresim/icanadolu.jpg). High quality figures are available online.

The Central Anatolian Region (Figure 2) is one of Turkey's seven geographical regions. The surface area is 151,000 square kilometers and it occupies 20% of Turkey's land area. It is the second largest geographical region after the Eastern Anatolian region. It is surrounded by other regions except the Southeastern Anatolian region. The Central Anatolian region contains the Aksaray, Ankara, Çankırı, Eskişehir, Karaman, Kayseri, Kırıkkale, Kırşehir, Konya, Nevşehir, Niğde, Sivas and Yozgat provinces. It is composed of Konya, Upper Sakarya, Middle Kızılırmak and Upper Kızılırmak subregions. The Central Anatolian region has an appearance that is simple in terms of landforms. Plains are usually found in most parts of the Central Anatolian region at 1000 m elevation. Areas and river valleys of the Sakarya and Kızıhrmak rivers are the lowest in elevation at about 700 meters. Volcanic mountains, which extend from Northeast to Southwest, are the Hasandağ, Karacadag, Karadağ, Erciyes and Melendiz mountains. Mountain chains include the Akdağlar, Hınzır, Tecer and Yıldız mountains. This Central Anatolian region is composed of many plateaus, including the western Haymana and Cihanbeyli plateaus, the southern Obruk plateau, the eastern Bozok (Kızılırmak) plateau, the Yazılıkaya (Bayat) plateau at the border with the Aegean Region and the Uzunyayla plateau at the border with the Eastern Anatolian region. The Salt Lake is the largest closed basin in the research area. Some of the plains of Central Anatolia are very broad. The Konya Plain is the largest plain of Turkey, which is at the base of an old lake. The Kızıhrmak, Sakarya, Porsuk, Çekerek and Delice are the most important rivers in the Central Anatolian region. The Salt Lake is the largest lake in the region. Other major lakes are the Akşehir, Eber, Ilgın (Çavuşçu), Tuzla, Seyfe, Eymir and Mogan.

High mountains surround the region. Humid and temperate sea air cannot penetrate into the Central Anatolian region, causing the summers to be hot and dry, and winters to be cold and snowy with a dominant continental climate. Elevation increases towards the eastern part of region and winter temperatures decrease to very low values. Precipitation is lowest at the Central Anatolian region of Turkey and it has 7% of all forests of Turkey (www.cografya.gen.tr/egitim/bolgeler/icanadolu.htm).

There are many studies on the Elateridae fauna of Turkey, which were done mostly by foreign researchers. Most of these studies are limited in scope and generally are descriptions of new species. Studies on Turkish Elateridae include the following: Bodemeyer (1900), Buysson (1891), Cate and Platia (1989, 1997), Cate et al. (2002), Cate (2007), Chassain (1998), Dolin (1995), Dolin and Mertlik (2002), Dušánek and Mertlik (2007), Fairmaire (1866), Guglielmi and Platia (1985), Gurjeva (1972, 1989), Gül-Zümreoğlu (1972), Gülperçin and Tezcan (2009), Heyden et al. (1906), Horion (1953), Jagemann (1939), Kabalak and Sert (2005), Kabalak and Sert (2006, 2009), Kesdek et al. (2006), Laibner (2000), Lodos (1998), Lohse (1979), Mertlik (2000, 2005), Mertlik and Dušánek (2006), Platia (1989, 1994, 2001, 2003a, 2003b, 2004a, 2004b), Platia and Cate (1990), Platia and Gudenzi (1996a, 1996b, 1998a, 1998b, 1999, 2000a, 2000b, 2002a, 2002b, 2004a, 2004b, 2005, 2007, 2009), Platia, Kabalak and Sert. (2007), Platia and Kovancı (2005), Platia and Marini (1990), Platia and Mertlik (1996), Platia and Schimmel (1991, 1992, 1993, 1994), Platia and Tarnawski (1998), Platia, Yıldırım and Kesdek (2007), Reitter (1905, 1910, 1911, 1918), Roubal (1924), Sahlberg (1912–1913), Schenkung (1925, 1927), Schimmel (1990), Schwarz (1892, 1900, 1902), Tarnawski (1995, 1996, 2001), Winkler (1924 – 1932), Wurst (1994, 1995a, 1995b, 1997), Wurst and Schimmel (1995), Yüksel (1970), Zeising and Brunne (2003).

The main aim of this research was to study the faunistic composition (species distributions of subfamilies and genera), ecological properties of species (abundance and rarity of species, vertical distributions of species, habitat preferences of species and seasonality of species and genera) and the zoogeographical composition of the Elateridae fauna including the faunal relations between the Central Anatolian region and other geographical regions of Turkey.

## Materials and Methods

In this study, species collected mostly between April and August between 2005–2008 from the Central Anatolian region were part of a Tübitak project entitled: “Systematic studies on the family Elateridae (Coleoptera) in Central Anatolian and Middle Black Sea regions of Turkey”. Some specimens from the insect collection of the Entomology Laboratory of the Department of Biology, Hacettepe University collected in 1995, 1999, 2001–2004 and 2009 were also included in this study. Specimens were collected using different methods (insect net, Japanese umbrella, light trap and aspirator). Locality details of specimens (collecting provinces and counties, GPS coordinates, altitudes, collecting dates) are given. In the Material Examined section, the collector's name is listed at the end as ‘col.’.

**Figure 3.**
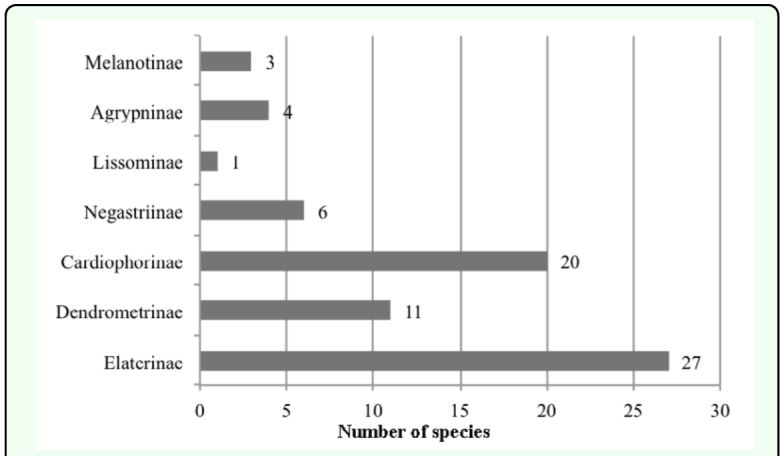
Number of species in subfamilies. High quality figures are available online.

**Figure 4.**
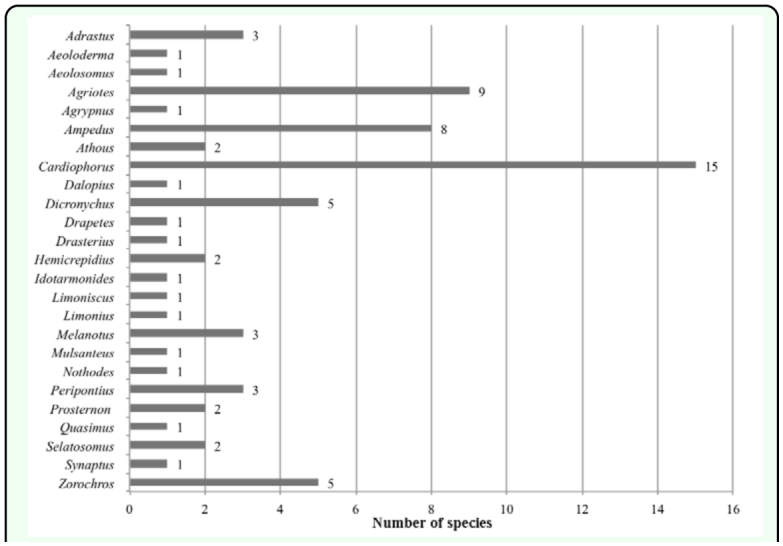
Number of species in genera. High quality figures are available online.

**Figure 5.**
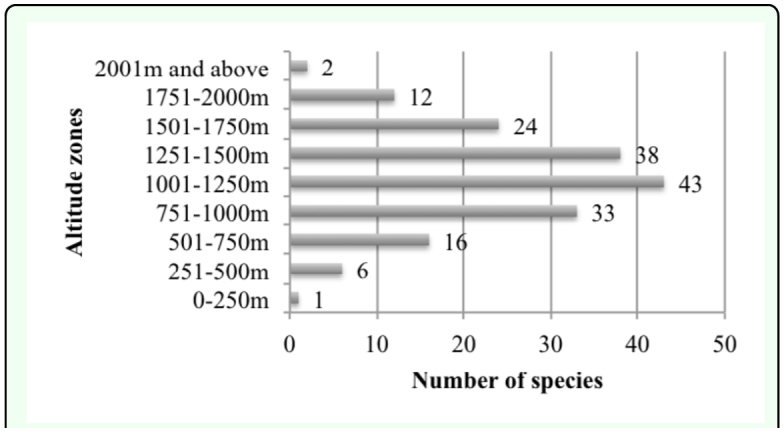
Number of collected species according to altitude zones in research area. High quality figures are available online.

**Figure 6.**
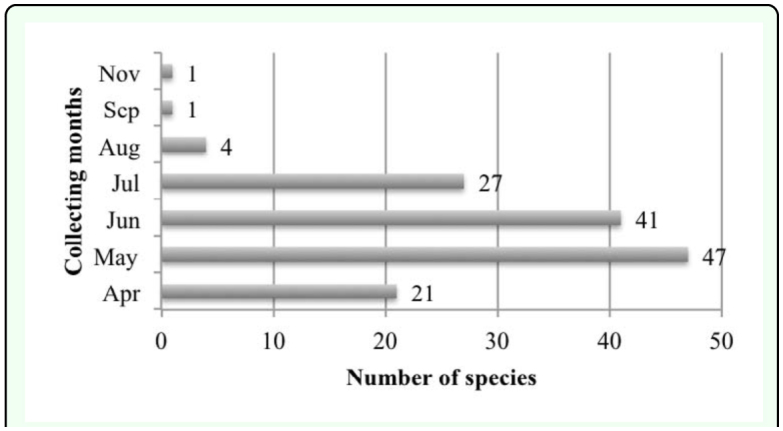
Number of species according to collecting months, **Apr:** April, **May:** May, **Jun:** June, **Jul:** July, **Aug:** August, **Sep:** September and **Nov:** November. High quality figures are available online.

**Figure 7.**
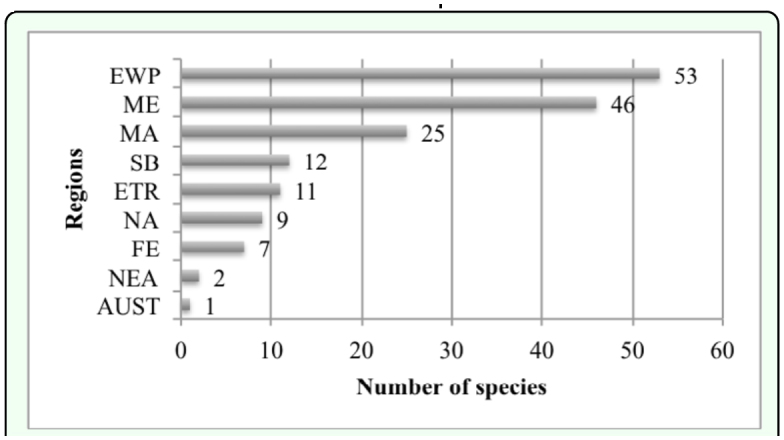
Distributions of detected species in Zoogeographical regions. **(ETR:** Endemic species for Turkey, **ME:** Middle East, **EWP:** European part of Western Palaearctic, **MA:** Middle Asia, **SB:** Siberia, **NA:** North Africa, **FE:** Far East, **AUST:** Australian Region, **NEA:** Nearctic Region) (Cate, 2007). High quality figures are available online.

**Figure 8.**
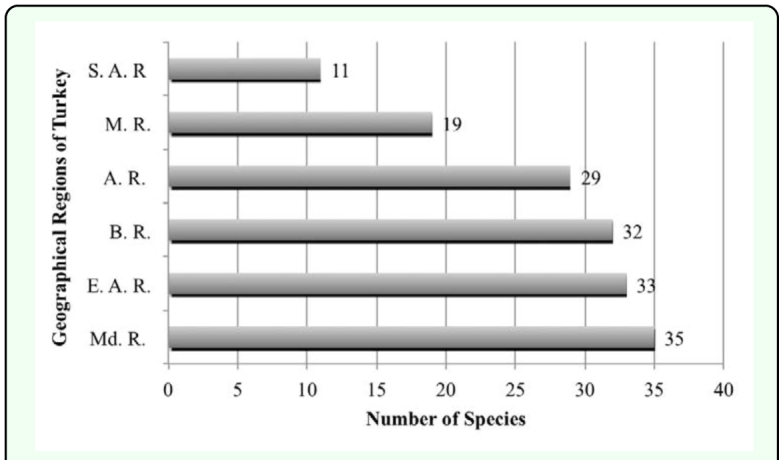
Number of shared species between research area and other geographical regions of Turkey. **S. A. R.:** Southeastern Anatolian Region, **M. R.:** Marmara Region, **A. R.:** Aegean Region, **E. A. R.:** Eastern Anatolian Region, **B. R.:** Blacksea Region, **Md. R.:** Mediterranean Region. High quality figures are available online.

Determinations of species were done by using published taxonomic keys (Gurjeva 1984; Laibner 2000; Platia 1994, 2003a, 2003c; Platia and Gudenzi 1998b, 2000b, 2002a, 2004a; Platia, Yıldırım and Kesdek 2007; Tarnawski 1995, 2000) and identifications were checked by Giuseppe Platia. Turkey and World distributions of species were taken from Cate (2007), Guglielmi and Platia (1985), Gülperçin and Tezcan (2009), Kabalak and Sert (2005), Kesdek et al. (2006), Laibner (2000), Mertlik and Dusanek (2006), Mertlik and Platia (2008), Platia (1994) and Platia and Gudenzi (2000a, 2002a). Classification system of the family Elateridae followed that of Mertlik and Platia (2008) except for the subfamily Lissominae for which we followed the concept of Laibner (2000) and Cate (2007). In the faunistic composition section, distributions of species in subfamilies and genera are shown (Figures 3 and 4) and species numbers of subfamilies and genera are compared between each other and species numbers of collected genera from the studied area are compared with known species numbers of genera recorded from Turkey. In the ecological properties of fauna section, specimen numbers of collected species, collecting habitats and methods, collecting months and vertical distributions of species (Figure 6) are given and assessed (Figures 5 and 6, Table 1). In the zoogeographical composition of fauna section, zoogeographical distributions and distributions in Turkey according to present literature of the identified species (Figures 7 and 8, Table 2) are given with information on endemic species. The Elateridae fauna of the studied area is compared with the Elateridae fauna of other geographical regions of Turkey. Zoogeographical distributions of identified species and the relationships between studied areas and zoogeographical regions are discussed.

**Table 1.**
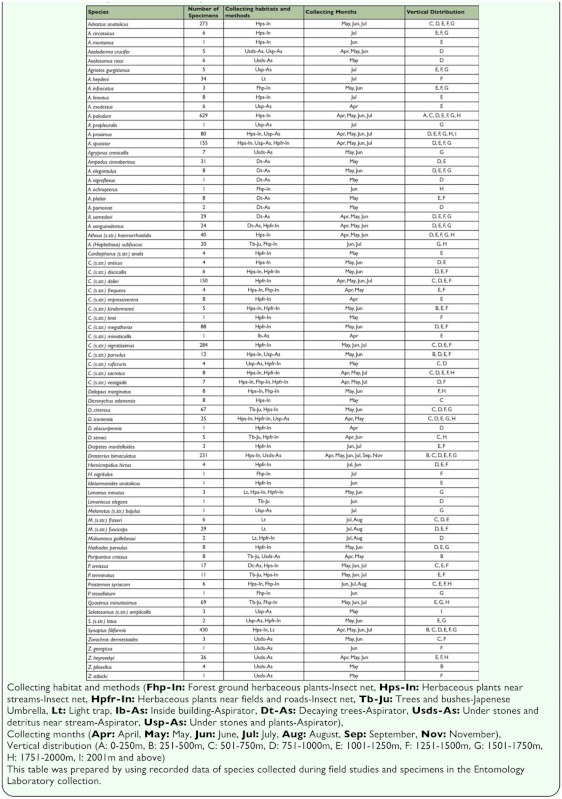
Number of species and collected specimens.

**Table 2.**
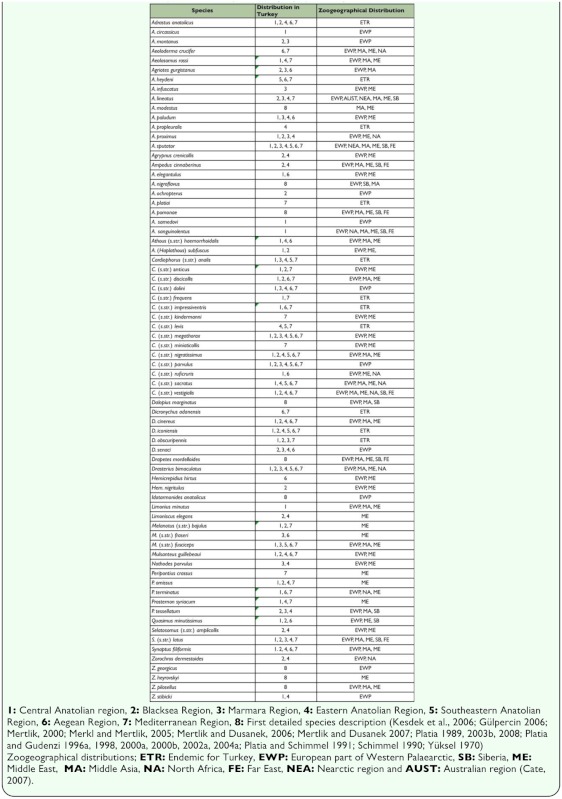
Distributions of detected species in Turkey and Zoogeographical regions

## Results and Discussion

### Annotated Checklist of Elateridae Species of the Central Anatolian Region of Turkey

**Subfamily: DENDROMETRINAE Gistel, 1856 Tribe: Athouini Candèze, 1859 Genus: *Nothodes* Le Conte , 1861 1. *Nothodes parvulus* (Panzer, 1799)**
Material Examined: **Çankırı:** Bayramören, 40°57′20″ N, 33°18′19″E, 936 m, 04.VI.2008, 1 specimen; **Kayseri:** Talas, 38°38′40″N, 35°36′17″E, 1424 m, 29.V.2007, 2 specimens; **Konya:** Beyşehir, 39°35′55″N, 31°26′49″E, 1124 m, 14.V.2005, 1 specimen; **Niğde:** Central County, 38°01′10″N, 35°01′09″E, 1573 m, 27.VI.2006, 1 specimen; **Yozgat:** Akdağmadeni, 39°43′28″N, 35°54′37″E, 1195 m, 12.VI.2007, 3 specimens; col. M. Kabalak.

**Genus: *Limonius* Eschscholtz, 1829. 2. *Limonius minutus* Linnaeus, 1758**
Material Examined: **Eskişehir:** Alpu 39°56′18″N, 31°08′34″E, 1515 m, 15.V.2007, 1 specimen; **Niğde:** Çamardı, 37°51′31″N, 34°57′30″E, 1580 m, 29.V.2005, 1 specimen; **Sivas:** Zara, 40°04′04″N, 37°43′39″E, 1626 m, 01.VI.2007, 1 specimen; col.M. Kabalak.

**Genus: *Limoniscus* Reitter, 1905. 3. *Limoniscus elegans* (Buysson, 1891)**
Material Examined: **Yozgat:** Çekerek, 40°08′43″N, 35°25′30″E, 945 m, 12.VI.2007, 1 specimen; col. M. Kabalak.

**Genus: *Athous* Eschscholtz, 1829. Subgenus: *Athous* Eschscholtz, 1829 4. *Athous* (s. str.) *haemorrhoidalis* (Fabricius, 1821)**
**Material Examined: Aksaray:** Ortaköy, 38°44′54″N, 33°59′55″E, 1256 m, 25.IV.2006, 2 specimens; **Çankırı:** Bayramören, 40°57′20″N, 33°18′19″E, 936 m, 04.VI.2008, 3 specimens; **Eskişehir:** Central County, 39°57′06″N, 30°31′03″E, 1045 m, 05.VI.2006, 1 specimen; **Niğde:** Çamardı, 37°48′52″N, 34°59′35″E, 1434 m, 23.V.2008, 4 specimens; Ulukışla, 37°26′50″N, 34°36′57″E, 1780 m, 22.V.2008, 4 specimens; Çamardı, 37°52′43″N, 35°06′25″E, 1512 m, 23.V.2008, 3 specimens; **Sivas:** Divriği, 39°30′27″N, 38°7′32″E, 1405 m, 22.V.2006, 3 specimens; Central County, 39°24′17″N, 36°41′25″E, 1366 m, 23.V.2006, 12 specimens; Gemerek, 39°18′18″N, 35°56′48″E, 1353 m, 30.V.2007, 6 specimens; Suşehri, 40°03′47″N, 38°03′10″E, 1356 m, 02.VI.2007, 1 specimen; **Yozgat:** Çandır, 39°15′13″N, 35°34′03″E, 1264 m, 11.VI.2007, 1 specimen; col. M. Kabalak.

**Subgenus: *Haplathous* Reitter, 1905  5. *Athous* (*H.*) *subfuscus* (O. F. Müller, 1764)**
Material Examined: **Çankırı:** Ilgaz 41°02′07″N, 33°47′28″E, 1502 m, 04.VI.2006, 5 specimens; Ilgaz 41°04′22″N, 33°43′47″E, 1851 m, 22.VII.2006, 3 specimens; Ilgaz 41°04′22″N, 33 43′47″E, 1867 m, 05.VI.2008, 4 specimens; Ilgaz 41°02′03″N, 33 47′31″E, 1639 m, 05.VI.2008, 5 specimens; **Sivas:** Koyulhisar, 40°21′06″N, 37°49′58″E, 1730 m, 02.VI.2007, 3 specimens; col. M. Kabalak.

**Tribe: Hemicrepidiini Champion, 1894 Genus: *Hemicrepidius* Germar, 1829. 6. *Hemicrepidius hirtus* (Herbst, 1784)**
Material Examined: **Yozgat:** Central County, 39°44′33″N, 34°48′15″E, 1097 m, 20.VII.2005, 1 specimen; Çandır, 39°14′50″N, 35°33′07″E, 1253 m, 12.VII.2006, 1 specimen; Central County, 39°41′41″N, 34°50′58″E, 1135 m, 11.VI.2007, 1 specimen; col. M. Kabalak. **Eskişehir:** Central County, 39°35′53″N, 30°16′42″E, 886 m, 02.VII.2009, 1 specimen; col. Y. Turan.

**7. *Hemicrepidius nigritulus* (Reitter, 1890) (Fig. 5.7 a–b).**
Material Examined: **Yozgat:** Sorgun 40°04′27″N, 35°14′41″E, 1442 m, 13.VII.2006, 1 specimen; col. M. Kabalak.

**Tribe: Ctenicerini Fleutiaux, 1936 Genus: *Prosternon* Latreille, 1834 8. *Prosternon syriacum* (Buysson, 1891).**
Material Examined: **Ankara:** Güdül 40°14′15″N, 32°15′40″E, 683 m, 09.VII.2003, 1 specimen; Kızılcahamam, 40°26′56″N, 32°50′41″E, 1464 m, 05.VIII.2008,1 specimen; **Çankırı:** Ilgaz, 41°04′22″N, 33°43′47″E, 1851 m, 22.VII.2006, 2 specimens; **Eskişehir:** İnönü 39°47′18″N, 30°05′41″E, 1123 m, 31.VII.2006, 1 specimen; col. M. Kabalak; **Eskişehir:** Mihalıççık, 39°59′13″N, 31°20 ′32″E, 643 m, 30.VI.2009, 1 specimen; col. Y. Turan.

**9. *Prosternon tessellatum* (Linnaeus, 1758)**
Material Examined: **Çankırı:** Ilgaz, 41°02′07″N, 33°47′28″E, 1502 m, 05.VI.2008, 1 specimen; col. M. Kabalak.

**Genus: *Selatosomus* Stephens, 1830 Subgenus: *Selatosomus* Stephens, 1830 10. *Selatosomus* (s. str.) *amplicollis* Dolin, 1982**
Material Examined: **Niğde:** Ulukışla, 37°24′52N, 34°33′55″E, 2341 m, 22.V.2008, 3 specimens; col. M. Kabalak.

**11. *Selatosomus* (s. str.) *latus* (Fabricius, 1801)**
Material Examined: **Nevşehir:** Ürgüp, 38°38′01″N, 34°53′52″E, 1151 m, 26.VI.2004, 1 specimen; **Yozgat:** Sorgun, 40°04′07″N, 35°15′23″E, 1545 m, 19.V.2008, 1 specimen; col. M. Kabalak.

**Subfamily: ELATERINAE Leach, 1815 Tribe: Elaterini Leach, 1815 Genus: *Mulsanteus* Gozis, 1875 12. *Mulsanteus guillebaeui* (Mulsant and Godart, 1853)**
Material Examined: **Ankara:** Yenimahalle, 39°57′58″N, 32°48′21″E, 885 m, 05.VIII.2002, col. N. Kocatepe, 1 specimen; **Nevşehir:** Gülşehir, 38°45′55″N, 34°36′00″E, 914 m, 06.VII.2006, 1 specimen; col. M. Kabalak.

**Tribe: Pomachiliini Candèze, 1859. Genus: *Idotarmonides* Agajev, 1985. 13. *Idotarmonides anatolicus* (Candèze, 1881)**
Material Examined: **Ankara:** Kızılcahamam 40°16′15″N, 32°25′30″E, 1188 m, 28.VI.2003, 1 specimen; col. M. Kabalak.

**Tribe: Synaptini Gistel, 1856. Genus: *Peripontius* Gurjeva, 1979 14. *Peripontius crassus* (Buyson, 1906)**
Material Examined: **Karaman:** Central County, 36°56′49″N, 33°02′20″E, 416 m, 28.V.2005, 6 specimens; Central County, 36°56′49″N, 33°02′20″E, 416 m, 22.IV.2006, 2 specimens; col. M. Kabalak.

**15. *Peripontius omissus* (Buyson, 1889)**
Material Examined: **Ankara:** Kazan 40°12′16″N, 32°34′47″E, 1064 m, 16.VI.2003,5 specimens; **Eskişehir:** Mihalıççık 39°59′12″N, 31°20 ′33″E, 646 m, 04.VI.2006, 2 specimens; **Kayseri:** Yahyalı, 38°02′27″N, 35°24′22″E, 1443 m, 08.VII.2006, 1 specimen; Develi, 38°11′51″N, 35°53′45″E, 1481 m, 29.V.2007, 1 specimen; **Konya:** Ahırlı, 37°11′48″N, 32°04′10″E, 1360 m, 10.VI.2006, 1 specimen; Bozkır, 37°06′10″N, 32°18′16″E, 1260 m, 10.VI.2006, 1 specimen; Hadim, 37°00′44″N, 32°19′52″E, 1380 m, 05.V.2007, 1 specimen; **Niğde:** Çamardı, 37°40,79200′N, 35°00,69900′E, 1232 m, 3 specimens; 12.V.2007; col. M. Kabalak. **Eskişehir:** Mihalgazi, 39°56′54″N, 30°40′06″E, 1166 m, 01.VII.2009, 1 specimen; **Konya:** Seydişehir, 37°27′35″N, 31°43′53″E, 1470 m, 03.VI.2009, 1 specimen; col. Y. Turan.

**16. *Peripontius terminatus* (Erichson, 1842)**
Material Examined: **Çankırı:** Şabanözü, 40°31′07″N, 33°26′34″E, 1187 m, 22.VII.2006, 2 specimens; **Konya:** Derebucak, 37°25′48″N, 31°30′52″E, 1253 m, 15.V.2005,1 specimen; Bozkır, 37°06′10″N, 32°18′16″E, 1260 m, 10.VI.2006, 1 specimen; Hadim, 37°00′44″N, 32°19′52″E, 1380 m, 05.V.2007, 7 specimens; col. M. Kabalak.

**Genus: *Adrastus* Eschscholtz, 1829 17. *Adrastus anatolicus* Platia and Schimmel, 1991**
Material Examined: **Aksaray:** Güzelyurt, 38°14′20″N, 34°16′47″E, 1324 m, 28.VI.2004, 2 specimens; Güzelyurt, 38°16′03″N, 34°17′16″E, 1194 m, 28.VI.2004, 1 specimen; **Kırşehir:** Mucur, 39°00′41″N, 34°28′19″E, 1071 m, 26.VI.2006; 1 specimen; col. T. Türkeş. **Aksaray:** Güzelyurt, 38°15′51″N, 34°17′25″E, 1197 m, 13.VI.2006, 43 specimens; **Ankara:** Kazan, 40°12′16″N, 32°34′47″E, 1064 m, 16.VI.2003, 7 specimens; Kızılcahamam, 40°20′00″N, 32°42′03″E, 971 m, 17.VI.2003, 1 specimen; Nallıhan, 40°13′53″N, 31°20′27″E, 658 m, 23.VI.2003, 9 specimens; Çubuk, 40°18′54″N, 32°55′46″E, 1072 m, 25.VI.2003, 5 specimens; Kızılcahamam, 40°18′43″N, 32°28′04″E, 792 m, 28.VI.2003, 3 specimens; Çamlıdere, 40°28′20″N, 32°20′45″E, 1037 m, 29.VI.2003, 1 specimen; Kızılcahamam, 40°41′00″N, 32°43′46″E, 1563 m, 10.VII.2003, 2 specimens; Kızılcahamam, 40°35′04″N, 32°39′41″E, 1068 m, 10.VII.2003, 44 specimens; **Çankırı:** Central County, 40°33′55″N, 33°33′25″E, 788 m, 03.VI.2006, 1 specimen; Ilgaz, 40°56′51″N, 33°39′45″E, 945 m, 22.VII.2006, 5 specimens; **Eskişehir:** Günyüzü, 39°20′41″N, 31°50′09″E, 859 m, 06.VII.2003, 1 specimen; **Kayseri:** Yeşilhisar, 38°21′34″N, 34°58′53″E, 1324 m, 07.VII.2006, 52 specimens; **Kırıkkale:** Çelebi, 39°28′37″N, 33°32′18″E, 1128 m, 22.VI.2007, 3 specimens; **Kırşehir:** Akpınar, 39°26′54″N, 33°58′58″E, 1157 m, 05.VII.2006, 1 specimen; Kaman, 39°27′47″N 33°36′31″E, 984 m, 22.VI.2007, 1 specimen; **Nevşehir:** Central County, 38°35′45″N 34°43′05″E, 1248 m, 07.VII.2006, 1 specimen; Ürgüp, 38°42′55″N, 35°00′31″E, 1077 m, 24.VI.2007, 9 specimens; Central County, 38°35′49″N, 34°43′05″E, 1254 m; 25.VI.2007, 4 specimens; **Niğde:** Çamardı, 37°52′43″N, 35°06′15″E, 1556 m, 21.VI.2006, 11 specimens; Çamardı, 37°48′40″N, 34°59′40″E, 1425 m, 21.VI.2006, 7 specimens; **Sivas:** Gürün, 38°45′29″N, 37°13′58″E, 1339 m, 31.V.2007, 1 specimen; **Yozgat:** Central County, 39°44′33″N, 34°48′15″E, 1241 m, 20.VII.2005, 14 specimens; Sorgun, 39°36′23″N, 35°15′25E, 1101 m, 11.VI.2007, 3 specimens; Central County, 39°41′41″N, 34°50′58″E, 1135 m, 11.VI.2007, 2 specimens; Central County, 39°56′14″N, 34°42′33″E, 1206 m, 13.VI.2007, 1 specimen; col. M. Kabalak. **Aksaray:** Güzelyurt, 38°15′13″N 34°18′07″E, 1266 m, 15.VI.2009, 2 specimens; Güzelyurt, 38°16′03″N, 34°17′16″E, 1194 m, 22.VI.2009, 14 specimens; **Ankara:** Kalecik, 40°11′41″N, 33°20′00″E, 1049 m, 11.VII.2008, 1 specimen; **Eskişehir:** Mihalıççık, 39°59′13″N, 31°20′32″E, 643 m, 30.VI.2009, 5 specimens; **Kırşehir:** Central County, 39°10′05″N, 33°55′39″E, 1039 m, 28.VI.2009, 3 specimens; **Nevşehir:** Ürgüp, 38°43′59″N, 34°55′50″E, 948 m, 24.VI.2009, 2 specimens; **Sivas:** Hafik, 39°55′12″N, 37°23′44″E, 1322 m, 18.VII.2009, 10 specimens; col. Y. Turan.

**18. *Adrastus circassicus* Reitter, 1896**
Material Examined: **Çankırı:** Kurşunlu, 40°52′03″N, 33°12′49″E, 1423 m, 27.VII.2005, 4 specimens; **Yozgat:** Central County, 39°38′07″N, 34°55′25″E, 1027 m, 12.VII.2006, 1 specimen; Akdağmadeni, 39°39′04″N, 35°57′37″E, 1628 m, 14.VII.2006, 1 specimen; col. M. Kabalak.

**19. *Adrastus montanus* (Scopoli, 1763)**
Material Examined: **Ankara:** Gölbaşı 39°35′56″N, 32°50′45″E, 1096 m, 24.VI.2003, 1 specimen; col. M. Kabalak.

**Genus: *Synaptus* Eschscholtz, 1829 20. *Synaptus filiformis* (Fabricius, 1781)**
Material Examined: **Aksaray:** Güzelyurt, 38°15′51″N, 34°17′25″E, 1197 m, 19.VI.2006, 18 specimens; Central County, 38°21′05″N, 34°13′34″E, 1116 m, 19.VI.2006, 1 specimen; Central County, 38°23′54″N, 34°07′21″E, 1071 m, 19.VI.2006, 2 specimens; Güzelyurt, 38°15′51″N, 34°17′27″E, 1301 m, 11.V.2007, 3 specimens; **Ankara:** Ayaş, 40°11′59″N, 32°27′51″E, 1036 m, 30.V.2004, 1 specimen; Ayaş, 40°06′03″N, 32°24′22″E, 1039 m, 30.V.2004, 5 specimens; **Çankırı:** Şabanözü, 40°31′07″N, 33°26′34″E, 1187 m, 22.VII.2006, 2 specimens; Şabanözü, 40°29′50″N, 33°24′38″E, 1178 m, 05.VI.2008, 6 specimens; Şabanözü, 40°24′13″N, 33°15′11″E, 923 m, 05.VI.2008, 1 specimen; **Eskişehir:** Mihalıççık, 39°59′12″N, 31°20′33″E, 646 m, 04.VI.2006, 2 specimens; Central County, 39°57′06″N, 30°31′03″E, 1045 m, 05.VI.2006, 1 specimen; Sarıcakaya, 39°57′02″N, 30°40′25″E, 1154 m, 30.VII.2006, 2 specimens; **Karaman:** Central County, 37°04′49″N, 33°05′31″E, 1285 m, 28.V.2005, 1 specimen; Central County, 36°47′04″N, 32°53′55″E, 1742 m, 28.V.2005, 2 specimens; Central County, 36°56′49″N, 33°02′20″E, 408 m, 28.V.2005, 1 specimen; Ermenek, 36°42′18″N, 32°57′18″E, 1483 m, 11.VI.2006, 2 specimens; Central County, 37°09′06″N, 33°33′07″E, 1206 m, 12.VI.2006, 4 specimens; **Kayseri:** Yeşilhisar, 38°21′34″N, 34°58′53″E, 1324 m, 07.VII.2006, 2 specimens; Yahyalı, 38°02′27″N, 35°24′22″E, 1443 m, 08.VII.2006, 1 specimen; Sarız, 38°21′55″N, 36°26′35″E, 1500 m, 09.VII.2006, 4 specimens; Özvatan, 39°5′17″N, 35°48′47″E, 1107 m, 11.VII.2006, 13 specimens; Central County, 38°47′57″N, 35°14′40″E, 1004 m, 12.VII.2006, 6 specimens; Sarıoğlan, 39°01′35″N, 36°03′27″E, 1229 m, 10.VII.2006, 3 specimens; Felahiye, 39°7′35.34″N, 35°38′7.86″E, 1401 m, 11.VII.2006, 7 specimens; Yahyalı, 38°03′15″N, 35°23′37″E, 1362 m, 28.V.2007, 1 specimen; Develi, 38°11′51″N, 35°53′45″E, 1481 m, 29.V.2007, 1 specimen; İncesu, 38°37′53″N, 35°09′28″E, 1117 m, 24.VI.2007, 4 specimens; **Kırşehir:** Akpınar, 39°21′47″N, 34°00′37″E, 1147 m, 04.VII.2006, 20 specimens; Akpınar, 39°26′54″N, 33°58′58″E, 1157 m, 05.VII.2006, 1 specimen; Akpınar, 39°26′43″N, 34°05′50″E, 1309 m, 05.VII.2006, 10 specimens; Central County, 39°30′33″N, 34°09′37″E, 1076 m, 09.VI.2007, 5 specimens; Central County, 38°18′15″N, 34°06′07″E, 1083 m, 09.VI.2007, 49 specimens; Central County, 39°19′18″N, 34°06′39″E, 1115 m, 22.VI.2007, 1 specimen; **Konya:** Akşehir, 38°18′24″N, 31°29′11″E, 1063 m, 13.V.2005, 6 specimens; Seydişehir, 37°23′33″N, 31°59′26″E, 1096 m, 18.IV.2006, 1 specimen; Derebucak, 37°25′48″N, 31°30′52″E, 1253 m, 15.V.2005, 5 specimens; Seydişehir, 37°27′56″N, 31°44′23″E, 1355 m, 15.V.2005, 2 specimens; Hadim, 37°06′28″N, 32°27′20″E, 1229 m, 27.V.2005, 2 specimens; Akşehir, 38°18′17″N, 31°27′57″E, 1081 m, 09.VI.2006, 3 specimens; Ilgın, 38°05′06″N, 31°49′32″E, 1193 m, 13.VI.2006, 5 specimens; Yunak, 38°55′03″N, 32°05′25″E, 926 m, 03.V.2007, 1 specimen; Sarayönü, 38°11′58″N, 32°25′49″E, 1147 m, 08.V.2007, 1 specimen; Hadim, 37°03′04″N, 32°35′29″E, 851 m, 06.V.2007, 1 specimen; **Nevşehir:** Ürgüp, 38°36′04″N, 34°54′24″E, 1092 m 07.VII.2006, 1 specimen; **Niğde:** Çamardı, 37°51′31″N, 34°57′30″E, 1580 m, 15.VII.2005, 13 specimens; Ulukışla, 37°27′10″N, 34°38′21″E, 1638 m, 20.VI.2006, 6 specimens; Çamardı, 37°42′50″N, 35°01′17″E, 1299 m, 21.VI.2006, 1 specimen; Çamardı, 37°48′40″N, 34°59′40″E, 1425 m, 21.VI.2006, 6 specimens; Çamardı, 37°42′49″N, 35°01′18″E, 1308 m, 23.V.2008, 4 specimens; Çamardı, 37°48′52″N, 34°59′35″E, 1434 m, 23.V.2008, 4 specimens; Central County, 38°00′46″N, 35°01′42″E, 1599 m, 23.V.2008, 2 specimens; **Sivas:** Doğanşar, 40°10′09″N, 37°34′21″E, 1385 m, 21.VII.2005, 6 specimens; Koyulhisar, 40°14′22″N, 37°58′21″E, 763 m, 21.VII.2005, 1 specimen; Suşehri, 40°03′01″N, 38°02′47″E, 1469 m, 21.VII.2005, 1 specimen; Divriği, 39°30′27″N, 38°07′32″E, 1405 m, 22.V.2006, 5 specimens; Gürün, 38°45′29″N, 37°13′59″E, 1364 m, 22.V.2006, 15 specimens; Central County, 39°38′28″N, 37°00′29″E, 1338 m, 22.V.2006, 6 specimens; Gemerek, 39°17′12″N, 36°00′25″E, 1187 m, 23.V.2006, 4 specimens; Yıldızeli, 39°49′51″N, 36°42′10″E, 1338 m, 24.V.2006, 2 specimens; Gemerek, 39°18′18″N, 35°56′48″E, 1345 m, 23.V.2006, 2 specimens; Şarkışla, 39°35′32″N, 36°09′38″E, 1458 m, 14.VII.2006, 1 specimen; Yıldızeli, 39°49′44″N, 36°42′15″E, 1341 m, 15.VII.2006, 2 specimens; İmranlı, 39°53′07″N, 38°01′52″E, 1568 m, 17.VII.2006, 5 specimens; Zara, 39°51′46″N, 37°45′40″E, 1337 m, 17.VII.2006, 2 specimens; Gürün, 38°45′29″N, 37°13′58″E, 1339 m, 01.V.2007, 13 specimens; Zara, 39°55′50″N, 37°50′26″E, 1620 m, 02.VI.2007, 1 specimen; Şarkışla, 39°34′27″N, 36°14′36″E, 1351 m, 03.VI.2007, 1 specimen; Yıldızeli, 39°49′38″N, 36°42′21″E, 1334 m, 04.VI.2007, 8 specimens; **Yozgat:** Yerköy, 39°36′47″N, 34°30′47″E, 760 m, 19.VII.2005, 1 specimen; Central County, 39°44′33″N, 34°48′15″E, 1241 m, 20.VII.2005, 1 specimen; Akdağmadeni, 39°40′51″N, 35°46′23″E, 1244 m, 20.VII.2005, 4 specimens; Akdağmadeni, 39°39′28″N, 36°00′43″E, 1380 m, 20.VII.2005, 5 specimens; Çayıralan, 39°17′05″N, 35°36′29″E, 1311 m, 12.VII.2006, 4 specimens; Central County, 39°38′7.68″N, 34°55′25.14″E, 1027 m, 12.VII.2006, 9 specimens; Central County, 39°32′53″N, 34°57′48″E, 953 m, 13.VII.2006, 4 specimens; Sarıkaya, 39°33′31″N, 35°34′00″E, 1145 m, 14.VII.2006, 2 specimens; Central County, 39°41′25″N, 34°38′10″E, 962 m, 10.VI.2007, 5 specimens; Çandır, 39°15′13″N, 35°34′03″E, 1264 m, 11.VI.2007, 13 specimens; Central County, 39°34′09″N, 34°59′41″E, 952 m, 11.VI.2007, 22 specimens; Akdağmadeni, 39°43′28″N, 35°54′37″E, 1195 m, 12.VI.2007, 2 specimens; col. M. Kabalak. **Aksaray:** Güzelyurt, 38°15′13″N, 34°18′07″E, 1266 m, 15.VI.2009, 11 specimens; Güzelyurt, 38°16′03″N, 34°17′16″E, 1196 m, 22.VI.2009, 2 specimens; **Ankara:** Beypazarı, 40°19′07″N, 31°59′56″E, 1135 m, 09.VII.2008, 1 specimen; Elmadağ, 39°44′03″N, 33°08′09″E, 1245 m, 30.V.2009, 2 specimens; **Eskişehir:** Mihalgazi, 39°56′54″N, 30°40′06″E, 1166 m, 01.VII.2009, 2 specimens; Central County, 39°35′53″N, 30°16′42″E, 886 m, 02.VII.2009, 1 specimen; **Karaman:** Central County, 37°09′12″N, 33°26′09″E, 1112 m, 12.VI.2009, 1 specimen; Başyayla, 36°45′15″N, 32°40′32″E, 1301 m, 13.VI.2009, 1 specimen; Central County, 36°50′38″N, 32°55′01″E, 1652 m, 13.VI.2009, 3 specimens; **Kırşehir:** Kaman, 39°12′49″N, 33°52′05″E, 1059 m, 28.VI.2009, 1 specimen; **Konya:** Akören, 37°31′42″N, 32°22′30″E, 1076 m, 04.VI.2009, 2 specimens; Ilgın, 38°03′37″N, 31°50′03″E, 1225 m, 05.VI.2009, 1 specimen; Akşehir, 38°18′18″N, 31°27′53″E, 1087 m, 05.VI.2009, 1 specimen; **Nevşehir:** Acıgöl, 38°41′22″N, 34°21′07″E, 1084 m, 25.VI.2009, 2 specimens; **Sivas:** Yıldızeli, 39°50′59″N, 36°42′51″E, 1325 m, 17.VII.2009, 1 specimen; Hafik, 39°55′12″N, 37°23′44″E, 1322 m, 18.VII.2009, 1 specimen; Doğanşar, 40°15′19″N, 37°32′45″E, 1069 m, 18.VII.2009, 2 specimens; Hafik, 40°02′02″N, 37°28′13″E, 1543 m, 18.VII.2009, 2 specimens; col. Y. Turan.

**Genus: *Ampedus* Dejean, 1833 21. *Ampedus cinnaberinus* (Eschscholtz, 1829)**
Material Examined: **Aksaray:** Gülağaç, 38°21′04″N, 34°13′32″E, 1056 m, 12.V.2007, 7 specimens; **Eskişehir:** Sivrihisar, 39°22′55″N, 31°29′28″E, 916 m, 14.V.2007, 2 specimens; **Karaman:** Central County, 37°07′13″N, 33°04′21″E, 1156 m, 06.V.2007, 4 specimens; **Kırıkkale:** Çelebi, 39°28′37″N, 33°32′18″E, 1128 m, 22.VI.2007, 6 specimens; **Konya:** Ilgın, 38°08′33″N, 31°48′34″E, 1069 m, 04.V.2007, 7 specimens; **Yozgat:** Akdağmadeni, 39°49′16″N, 35°53′50″E, 1219 m, 06.V.2006, 5 specimens; col. M. Kabalak.

**22. *Ampedus elegantulus* (Schönherr, 1817)**
Material Examined: **Aksaray:** Güzelyurt, 38°16′27″N, 34°22′42″E, 1538 m, 11.V.2007, 3 specimens; **Çankırı:** Central County, 40°38′05″N, 33°36′28E, 754 m, 05.VI.2008, 1 specimen; **Karaman:** Central County, 37°12′03″N, 33°24′10″E, 1069 m, 07.V.2007, 1 specimen; **Konya:** Derbent, 37°53′00″N, 31°59′42″E, 1305 m, 04.V.2007, 3 specimens; col. M. Kabalak.

**23. *Ampedus nigroflavus* (Goeze, 1777)**
Material Examined: **Yozgat:** Şefaatli, 39°30′19″N, 34°44′34″E, 915 m, 05.V.2006, 1 specimen; col. M. Kabalak.

**24. *Ampedus ochropterus* Germar, 1844**
Material Examined: **Çankırı:** Ilgaz, 41°03′57″N, 33°44′47″E, 1866 m, 05.VI.2008, 1 specimen; col. M. Kabalak.

**25. *Ampedus platiai* Schimmel, 1990**
Material Examined: **Konya:** Selçuklu, 37°55′18″N. 32°16′17″E. 1275 m, 04.V.2007, 2 specimens; **Yozgat:** Central County, 39°38′07″N, 34°55′21″E, 1040 m, 04.V.2006, 5 specimens; Çayıralan, 39°17′06″N, 35°36′30″E, 1309 m. 05.V.2006, 1 specimen; col. M. Kabalak.

**26. *Ampedus pomonae* (Stephens, 1830)**
Material Examined: **Yozgat:** Çekerek, 40°05′13″N, 35°35′14″E, 775 m, 06.V.2006, 2 specimens; col. M. Kabalak.

**27. *Ampedus samedovi* Dolin and Agajev, 1983**
Material Examined: **Aksaray:** Ortaköy, 38°44′54″N, 33°59′55″E, 1256 m, 25.IV.2006, 5 specimens; **Kayseri:** Felahiye, 39°07′16″N, 35°37′47″E, 1388 m, 04.V.2006, 5 specimens; **Kırıkkale:** Çelebi, 39°31′09″N, 33°29′56″E, 895 m, 07.V.2006, 6 specimens; Keskin, 39°46′45″N, 33°55′17″E, 772 m, 07.V.2006, 1 specimen; **Nevşehir:** Central County, 38°37′04″N, 34°47′15″E, 1329 m, 30.IV.2006, 2 specimens; **Niğde:** Central County, 38°00′52″N, 35°01′30″E, 1589 m, 12.V.2007, 3 specimens; **Sivas:** Central County, 39°38′28″N, 37°00′29″E, 1338 m, 22.V.2006, 4 specimens; Zara, 40°00′01″N, 37°43′49″E 1559m 1 specimen 01.VI.2007; **Yozgat:** Central County 39°37′44″N 34°52′07″E, 1149 m, 05.V.2006, 2 specimens; col. M. Kabalak.

**28. *Ampedus sanguinolentus* (Schrank, 1776)**
Material Examined: **Aksaray:** Ortaköy, 38°44′54″N, 33°59′55″E, 1256 m, 25.IV.2006, 2 specimens; Ortaköy, 38°44′53″N, 33°59′56″E, 1257 m, 11.V.2007, 2 specimens; **Eskişehir:** Mahmudiye, 39°33′26N, 30°50′37″E, 917 m, 16.V.2007, 1 specimen; **Karaman:** Central County, 37°12′03″N, 33°24′10″E, 1069 m, 07.V.2007, 2 specimens; **Kayseri:** Tomarza, 38°24′27″N, 35°57′5″E, 1370 m, 02.V.2006, 2 specimens; Özvatan, 39°08′03″N,, 35°38′57″E, 1414 m, 30.V.2007, 1 specimen; **Konya:** Ilgın, 38°11′00″N, 31°50′45″E, 1092 m, 17.IV.2006, 1 specimen; Sarayönü, 38°11′58″N, 32°25′49″E, 1147 m, 08.V.2007, 1 specimen; **Niğde:** Central County, 38°00′52″N, 35°01′30″E, 1589 m, 12.V.2007, 3 specimens; Çamardı, 37°48′52″N, 34°59′35″E, 1434 m, 23.V.2008,1 specimen; **Sivas:** Central County, 39°38′28″N, 37°0′29″E, 1338 m, 22.V.2006, 1 specimen ;Yıldızeli, 39°49′51″N, 36°42′10″E, 1338 m, 24.V.2006, 1 specimen; Yıldızeli, 39°49′51″N, 36°42′10″E, 1338 m, 24.V.2006, 3 specimens; **Yozgat:** Şefaatli, 39°30′19″N, 34°44′34″E, 915 m. 05.V.2006, 1 specimen; Central County, 39°37′44″N, 34°52′7″E. 1149 m, 05.V.2006, 1 specimen; Çandır, 39°15′13″N, 35°34′03″E, 1264 m, 11.VI.2007, 1 specimen; col. M. Kabalak.

**Tribe: Agriotini Champion, 1894 Genus: *Dalopius* Eschscholtz, 1829 29. *Dalopius marginatus* (Linnaeus, 1758)**
Material Examined: **Sivas:** Gemerek, 39°18′18″N, 35°56′48″E, 1345 m, 23.V.2006, 2 specimens; Gemerek. 39°28′40″N, 35°57′59″E, 1809 m, 03.VI.2007, 6 specimens; col. M. Kabalak.

**Genus: *Agriotes* Eschshcoltz, 1829 30. *Agriotes gurgistanus* (Faldermann, 1835)**
Material Examined: **Kayseri:** Pınarbaşı, 38°48′41″N, 35°58′37″E, 1609 m, 20.VII.03, 3 specimens; **Yozgat:** Saraykent, 39°40′23″N. 35°30′29″E, 1145 m, 18.VII.2003, 1 specimen; col. N. Kocatepe; **Kayseri:** Sarıoğlan, 39°09′38″N, 35°51′41″E, 1398 m, 21.VII.09, 1 specimen; col. Y. Turan.

**31. *Agriotes heydeni* Schwarz, 1891**
Material Examined: **Sivas:** Central County, 39°41′57″N, 37°00′33″E, 1255 m, 20.VII.2005, 13 specimens; Central County, 39°41′57″N, 37°00′33″E, 1255 m, 21.VII.2005, 13 specimens; Central County, 39°41′57″N, 37°00′33″E, 1255 m, 15.VII.2006, 8 specimens; col. M. Kabalak.

**32. *Agriotes infuscatus* Desbrochers des Loges, 1870**
Material Examined: **Ankara:** Çubuk, 40°57′38″N, 33°06′32″E, 1085 m, 13.VI.2008, 1 specimen; **Çankırı:** Ilgaz, 41°02′03″N, 33°47′31″E, 1639 m, 05.VI.2008, 1 specimen; **Kayseri:** Özvatan, 39°08′03″N, 35°38′57″E, 1414 m, 30.V.2007, 1 specimen; col. M. Kabalak.

**33. *Agriotes lineatus* (Linnaeus, 1767)**
Material Examined: **Ankara:** Kızılcahamam, 40°34′58″N, 32°39′44″E, 1067 m, 10.VII.2003, 8 specimens; col. M. Kabalak.

**34. *Agriotes modestus* Kiesenwetter, 1858**
Material Examined: **Kayseri:** Develi, 38°23′21″N, 35°21′52″E, 1075 m, 12.IV.2003, 6 specimens; col. M. Kabalak.

**35. *Agriotes paludum* Kiesenwetter, 1859**
Material Examined: **Ankara:** Beytepe 39°51′57″N, 32°44′25″E, 1050 m, 18.V.1995, col. H. Alakoç, 1 specimen. **Aksaray:** Ortaköy, 38°44′54″N, 33°59′55″E, 1256 m, 19.VI.2006, 1 specimen; **Ankara:** Çankaya, 39°51′57″N, 32°44′25″E, 1050 m, 25.VI.2002, 1 specimen; Çankaya, 39°52′40″N, 32°43′49″E, 1050 m, 11.VII.2002, 1 specimen; Elmadağ, 39°52′55″N, 33°16′38″E, 981 m, 15.V.2003, 7 specimens; Güdül, 40°09′16″N, 32°14′06″E, 1036 m, 24.V.2003, 2 specimens; Güdül, 40°16′52″N, 32°07′17″E, 844 m, 24.V.2003, 15 specimens; Haymana, 39°26′03″N, 32°27′10″E, 1198 m, 25.V.2003, 1 specimen; Evren, 38°59′39″N, 33°43′44″E, 1150 m, 28.V.2003, 16 specimens; Akyurt, 40°08′45″N, 33°14′42″E, 1179 m, 30.V.2003, 3 specimens; Beypazarı, 40°19′12″N, 32°02′22″E, 1193 m, 07.VI.2003, 1 specimen; Elmadağ, 39°58′21″N, 33°09′37″E, 1123 m, 08.VI.2003, 1 specimen; Yenimahalle, 40°07′50″N, 32°45′41″E, 1084 m, 16.VI.2003, 2 specimens; Kazan, 40°12′16″N, 32°34′47″E, 1064 m, 16.VI.2003, 7 specimens; Kızılcahamam, 40°20′00″N, 32°42′02″E, 971 m, 17.VI.2003, 33 specimens; Polatlı, 39°22′39″N, 32°06′52″E, 862 m, 18.VI.2003, 1 specimen; Polatlı, 39°43′27″N, 32°05′00″E, 838 m, 18.VI.2003, 1 specimen; Polatlı, 39°26′55″N, 32°07′21″E, 816 m, 18.VI.2003, 9 specimens; Nallıhan, 40°17′02″N, 31°28′22″E, 957 m, 23.VI.2003, 30 specimens; Çubuk, 40°18′53″N, 32°55′46″E, 1073 m, 25.VI.2003, 21 specimens; Kızılcahamam, 40°18′44″N, 32°28′09″E, 795 m, 28.VI.2003, 4 specimens; Güdül, 40°05′51″N, 32°15′04″E, 673 m, 09.VII.2003, 3 specimens; Mamak, 39°56′39″N, 33°02′08″E, 997 m, 25.VII.2003, 7 specimens; Güdül, 40°10′55″N, 32°20′19″E, 1007 m, 30.V.2004, 1 specimen; Kalecik, 40°14′24″N, 33°27′28″E, 842 m, 09.V.2006, 8 specimens; Kalecik, 40°11′28″N, 33°19′52″E, 1049 m, 09.V.2006, 2 specimens; Çankaya, 39°52′40″N, 32°43′49″E, 1050 m, 22.IV.2008, 35 specimens; **Çankırı:** Ilgaz, 40°56′51″N, 33°39′45″E, 945 m, 22.VII.2006, 1 specimen; Ilgaz, 40°53′15″N, 33°30′22″E, 947 m, 27.VII.2005, 2 specimens; Şabanözü, 40°24′13″N, 33°15′11″E, 923 m, 05.VI.2008, 3 specimens; **Eskişehir:** Günyüzü, 39°20′38″N, 31°50′04″E, 873 m, 06.VII.2003, 1 specimen; Mihalıççık, 39°59′12″N, 31°20′33″E, 646 m, 04.VI.2006, 6 specimens; Seyitgazi, 39°28′50″N, 30°39′42″E, 1000 m, 05.VI.2006, 1 specimen; Günyüzü, 39°21′15″N, 31°50′38″E, 834 m, 29.VII.2006, 1 specimen; Seyitgazi, 39°21′26″N, 30°47′04″E, 996 m, 29.VII.2006, 2 specimens; İnönü, 39°49′15″N, 30°10′06″E, 818 m, 30.VII.2006, 1 specimen; Günyüzü, 39°10′04″N, 31°38′47″E, 847 m, 13.V.2007, 1 specimen; Seyitgazi, 39°28′50″N, 30°39′42″E, 1000 m, 14.V.2007, 2 specimens; Seyitgazi, 39°21′26″N, 30°47′04″E, 996 m, 16.V.2007, 1 specimen; Mihalıççık, 39°59′12″N, 31°20′33″E, 646 m, 04.VI.2006, 23 specimens; **Karaman:** Ermenek, 37°04′49″N, 33°05′31″E, 1285 m, 28.V.2005, 1 specimen; Central County, 37°09′06″N, 33°33′07″E, 1206 m, 12.VI.2006, 2 specimens; Central County, 37°07′13″N, 33°04′21″E, 1156 m, 06.V.2007, 16 specimens; Central County, 37°12′03″N, 33°24′10″E, 1069 m, 07.V.2007, 1 specimen; **Kayseri:** Central County, 38°50′57″N, 36°19′40″E 1682 m, 12.VII.2006, 3 specimens; Central County, 38°55′10″N, 35°04′59″E, 1210 m, 27.V.2007, 4 specimens; Felahiye, 39°03′42″N, 35°33′00″E, 1225 m, 30.V.2007, 2 specimens; Özvatan, 39°08′03″N, 35°38′57″E, 1414 m, 30.V.2007, 1 specimen; İncesu, 38°37′53″N, 35°09′28″E, 1117 m, 24.VI.2007, 17 specimens; **Kırıkkale:** Keskin, 39°46′45″N, 33°55′17″E, 772 m, 07.V.2006, 7 specimens; Sulakyurt, 40°19′24″N, 33°47′38″E, 571 m, 07.V.2006, 1 specimen; Çelebi, 39°28′37″N, 33°32′18″E, 1128 m, 22.VI.2007, 2 specimens; **Kırşehir:** Kaman, 39°25′59″N, 33°45′20″E, 974 m, 09.VI.2007, 2 specimens; Central County, 39°18′15″N, 34°06′07″E, 1083 m, 09.VI.2007, 2 specimens; **Konya:** Akşehir, 38°26′50″N, 31°30′58″E, 952 m, 13.V.2005, 17 specimens; Akşehir, 38°18′24″N, 31°29′11″E, 1063 m, 13.V.2005, 1 specimen; Doğanhisar, 38°09′26″N, 31°39′41″E, 1196 m, 14.V.2005, 1 specimen; Beyşehir, 39°35′55″N, 31°26′49″E, 1124 m, 14.V.2005, 34 specimens; Seydişehir, 37°27′56″N, 31°44′23″E, 1355 m, 15.V.2005, 5 specimens; Tuzlukçu, 38°26′31″N, 31°39′53″E, 1002 m, 16.IV.2006, 4 specimens; Ilgın, 38°16′23″N, 31°42′25″E, 1101 m, 17.IV.2006, 5 specimens; Beyşehir, 37°38′27″N, 31°37′44″E, 1128 m, 18.IV.2006, 1 specimen; Seydişehir, 37°23′33″N, 31°59′26″E, 1096 m, 18.IV.2006, 3 specimens; Akşehir, 38°18′17″N, 31°27′57″E 1081 m, 09.VI.2006, 6 specimens; Bozkır, 37°06′10″N, 32°18′16″E, 1260 m, 10.VI.2006, 2 specimens; Selçuklu, 37°55′20″N, 32°16′12″E, 1269 m, 13.VI.2006, 5 specimens; Ilgın, 38°05′06″N, 31°49′32″E, 1193 m, 13.VI.2006, 17 specimens; Akşehir, 38°26′57″N, 31°31′02″E, 967 m, 03.V.2007, 1 specimen; Hadim, 37°00′44″N, 32°19′52″E, 1380 m, 05.V.2007, 1 specimen; Hadim, 37°03′04″N, 32°35′29″E, 851 m, 06.V.2007, 3 specimens; Sarayönü, 38°11′58″N, 32°25′49″E, 1147 m, 08.V.2007, 1 specimen; **Nevşehir:** Gülşehir, 38°45′49″N, 34°35′43″E, 949 m, 29.IV.2006, 1 specimen; Ürgüp, 38°42′55″N, 35°00′31″E, 1077 m, 24.VI.2007, 8 specimens; Ürgüp, 38°33′39″N, 34°55′18″E, 1169 m, 24.VI.2007, 9 specimens; **Niğde:** Ulukışla, 37°26′50″N, 34°36′57″E, 1780 m, 22.V.2008, 2 specimens; **Sivas:** Suşehri, 40°17′17″N, 38°09′52″E, 966 m, 16.VII.2006, 2 specimens; Gürün, 38°45′29″N, 37°13′58″E, 1339 m, 31.V.2007, 3 specimens; Divriği, 39°17′26″N, 38°01′43″E, 1185 m, 01.VI.2007, 5 specimens; Suşehri, 40°15′40″N, 38°09′27″E, 816 m, 02.VI.2007, 8 specimens; **Yozgat:** Central County, 39°38′41″N, 34°54′58″E, 1034 m, 19.VII.2005, 21 specimens; Central County, 39°44′33″N, 34°48′15″E, 1097 m, 20.VII.2005, 10 specimens; Central County, 39°38′07″N, 34°55′21″E, 1040 m, 04.V.2006, 1 specimen; Şefaatli, 39°30′19″E, 34°44′34″E, 915 m, 05.V.2006, 2 specimens; Çekerek, 40°05′13″N, 35°35′14″E, 775 m, 06.V.2006, 2 specimens; Şefaatli, 39°31′46″N, 34°52′59″E, 958 m, 13.VII.2006, 3 specimens; Şefaatli, 39°31′40″N, 34°43′04″E, 902 m, 13.VII.2006, 3 specimens; Central County, 39°32′53″N, 34°57′48″E, 953 m, 13.VII.2006, 12 specimens; Çekerek, 40°05′41″N, 35°28′30″E, 1013 m, 14.VII.2006, 4 specimens; Central County, 39°41′25″N, 34°38′10″E, 962 m, 10.VI.2007, 33 specimens; Central County, 39°38′07″N, 34°55′22″E, 1043 m, 11.VI.2007, 8 specimens; Central County, 39°36′29″N, 34°58′06″E, 982 m, 11.VI.2007, 3 specimens; Central County, 39°34′09″N, 34°59′41″E, 952 m, 11.VI.2007, 28 specimens; Akdağmadeni, 39°43′28″N, 35°54′37″E, 1195 m, 12.VI.2007, 3 specimens; Central County, 39°56′14″N, 34°42′33″E, 1206 m, 13.VI.2007, 1 specimen; col. M. Kabalak. **Ankara:** Şereflikoçhisar, 39°05′19″N, 33°32′06″E, 947 m, 25.V.2008, 4 specimens; Ayaş, 40°00′12″N, 32°20′37″E, 960 m, 14.VI.2008, 1 specimen; **Eskişehir:** Sarıcakaya, 40°05′22″N, 30°50′38″E, 250 m, 01.VII.2009, 1 specimen; Central County, 39°34′50″N, 30°16′40″E, 911 m, 02.VII.2009, 1 specimen; **Karaman:** Central County, 37°09′11″N, 33°26′09″E, 1112 m, 12.VI.2009, 2 specimens; **Kırşehir:** Akpınar, 39°31′12″N, 33°59′53″E, 941 m, 28.VI.2009, 2 specimens; **Konya:** Ilgın, 38°03′37″N, 31°50′03″E, 1225 m, 05.VI.2009, 16 specimens; **Nevşehir:** Ürgüp, 38°32′53″N, 34°55′17″E, 1223 m, 24.VI.2009, 3 specimens; col. Y. Turan.

**36. *Agriotes propleuralis* Platia and Gudenzi, 1998**
Material Examined: **Kayseri:** Pınarbaşı, 38°42′28″N, 36°26′07″E, 1547 m, 20.VII.2003, 1 specimen; col. N. Kocatepe.

**37. *Agriotes proximus* Schwarz, 1891**
Material Examined: **Aksaray:** Ortaköy, 38°42′56″N, 34°06′14″E, 1143 m, 11.V.2007; 1 specimen; **Ankara:** Ayaş, 40°11′59″N, 32°27′51″E, 1036 m, 30.V.2004, 1 specimen; Çankaya, 39°51′57″N, 32°44′25″E, 1050 m; 22.IV.2008, 4 specimens; **Çankırı:** Korgun, 40°49′01″N, 33°33′28″E, 1229 m, 28.VII.2005, 1 specimen; **Eskişehir:** Seyitgazi, 39°28′50″N, 30°39′42″E, 1000 m, 05.VI.2006, 1 specimen; Seyitgazi, 39°21′26″N, 30°47′04″E, 996 m, 29.VII.2006, 1 specimen; Mahmudiye, 39°33′26″N, 30°50′37″E, 917 m, 16.V.2007, 1 specimen; **Kayseri:** Tomarza, 38°25′25″N, 35°51′38″E, 1364 m, 02.V.2006, 1 specimen; Bünyan, 38°51′24″N, 35°47′11″E, 1233 m, 10.VII.2006, 1 specimen; Sarız, 38°21′55″N, 36°26′35″E, 1500 m, 09.VII.2006, 1 specimen; Sarız, 38°27′00″N, 36°28′42″E, 1559 m, 29.V.2007, 8 specimens; Develi, 38°11′51″N, 35°53′45″E, 1481 m, 29.V.2007, 1 specimen; Özvatan, 39°08′03″N, 35°38′57″E, 1414 m, 30.V.2007, 3 specimens; Pınarbaşı, 38°54′17″N, 36°26′47″E, 1566 m, 31.V.2007, 1 specimen; Pınarbaşı, 38°50′57″N, 36°19′40″E, 1682 m, 31.V.2007, 7 specimens; Pınarbaşı, 38°49′11″N, 36°11′12″E, 1552 m, 31.V.2007, 1 specimen; **Kırşehir:** Akpınar, 39°21′47″N, 34°00′37″E, 1147 m, 04.VII.2006, 1 specimen; Akpınar, 39°26′43″N, 34°05′50″E, 1309 m, 05.VII.2006, 1 specimen; Kaman, 39°21′55″N, 33°47′14″E, 1049 m, 09.VI.2007, 1 specimen; Central County, 39°18′15″N, 34°06′07″E, 1083 m, 09.VI.2007, 2 specimens; Akçakent, 39°37′02″N, 34°11′55″E, 1387 m, 09.VI.2007, 1 specimen; **Konya:** Beyşehir, 39°35′55″N, 31°26′49″E, 1124 m, 14.V.2005, 1 specimen; Çumra, 37°32′33″N, 32°40′32″E, 1025 m, 27.V.2005, 1 specimen; Selçuklu, 37°55′20″N, 32°16′12″E, 1269 m, 13.VI.2006, 1 specimen; Seydişehir, 37°27′25″N, 31°42′39″E, 1727 m, 05.V.2007, 3 specimens; **Niğde:** Ulukışla, 37°24′51″N, 34°33′55″E, 2341 m, 22.V.2008, 3 specimens; **Sivas:** Yıldızeli, 39°49′44″N, 36°42′16″E, 1339 m, 22.VII.2005, 3 specimens; Divriği, 39°30′27″N, 38°07′32″E, 1405 m, 22.V.2006, 1 specimen; Central County, 39°38′28″N, 37°00′29″E, 1338 m, 22.V.2006, 1 specimen; Yıldızeli, 39°49′51″N, 36°42′10″E, 1338 m, 24.V.2006, 2 specimens; Yıldızeli, 39°49′44″N, 36°42′15″E, 1341 m, 15.VII.2006, 6 specimens; İmranlı, 39°53′07″N, 38°1′52″E, 1568 m, 17.VII.2006, 1 specimen; Zara, 40°00′01″N, 37°43′49″E, 1559 m, 01.VI.2007, 1 specimen; Zara, 40°04′04″N, 37°43′39″E, 1692 m, 01.VI.2007, 1 specimen; Zara, 40°04′55″N, 37°43′41″E, 1571 m, 01.VI.2007, 1 specimen; Şarkışla, 39°34′27″N, 36°14′36″E, 1351 m, 03.VI.2007, 1 specimen; Şarkışla, 39°33′38″N, 36°17′36″E, 1299 m, 03.VI.2007, 1 specimen; Gemerek, 39°28′40″N, 35°57′59″E, 1809 m, 03.VI.2007, 1 specimen; **Yozgat:** Sarıkaya, 39°26′34″N, 35°21′38″E, 1185 m, 04.V.2006, 2 specimens; Central County, 39°37′44″N, 34°52′07″E, 1149 m, 05.V.2006, 1 specimen; Çekerek, 40°05′41″N, 35°28′30″E, 1013 m, 14.VII.2006, 2 specimens; Çandır, 39°15′13″N, 35°34′03″E, 1264 m, 11.VI.2007, 2 specimens; Akdağmadeni, 39°32′56″N, 35°41′33″E, 1348 m, 13.VI.2007, 1 specimen; col. M. Kabalak. **Kayseri:** Develi, 38°25′28″N, 35°17′42″E, 1086 m, 02.VII.2003, col. N. Yanbuloğlu, 1 specimen. **Ankara:** Ayaş, 40°01′43″N, 32°22′39″E, 1087 m, 29.V.2008, 1 specimen; **Sivas:** Hafik, 39°55′12″N, 37°23′44″E, 1322 m, 18.VII.2009, 1 specimen; col. Y. Turan.

**38. *Agriotes sputator* (Linnaeus, 1758)**
Material Examined: **Ankara:** Kızılcahamam, 40°27′05″N, 32°37′17″E, 1216 m, 14.V.1995, 1 specimen; Çankaya, 39°52′17″N, 32°44′08″E, 1042 m, 18.V.1995, 1 specimen; Çankaya, 39°52′17″N, 32°44′08″E, 1042 m, 24.IV.2002, 4 specimens; Çankaya, 39°52′17″N, 32°44′08″E, 1042 m, 11.VII.2002, 1 specimen; Haymana, 39°32′39″N, 32°38′55″E, 1150 m, 10.V.2003, 23 specimens; Bala, 39°38′31″N, 33°03′31″E, 969 m, 15.V.2003, 1 specimen; Bala, 39°38′32″N, 33°03′33″E, 970 m, 15.V.2003, 2 specimens; Çubuk, 40°17′02″N, 33°02′50″E, 1193 m, 16.V.2003, 28 specimens; Kızılcahamam, 40°37′53″N, 32°29′28″E, 1379 m, 21.V.2003, 1 specimen; Kızılcahamam, 40°38′22″N, 32°30′24″E, 1364 m, 21.V.2003, 1 specimen; Kızılcahamam, 40°39′09″N, 32°36′38″E, 1590 m, 21.V.2003, 2 specimens; Haymana, 39°27′31″N, 32°25′42″E, 1124 m, 25.V.2003, 3 specimens; Evren, 38°59′39″N, 33°43′44″E, 1150 m, 28.V.2003, 4 specimens; Çubuk, 40°16′50″N, 33°00′27″E, 1156 m, 29.V.2003, 6 specimens; Elmadağ, 39°48′48″N, 33°07′58″E, 1191,m, 08.VI.2003, 1 specimen; Gölbaşı, 39°35′56″N, 32°50′45″E, 1096 m, 24.VI.2003, 1 specimen; Çamlıdere, 40°30′14″N, 32°25′40″E, 1183 m, 29.VI.2003, 1 specimen; Güdül, 40°10′55″N, 32°20′19″E, 1007 m, 30.V.2004, 3 specimens; Ayaş, 40°06′03″N, 32°24′22″E, 1039 m, 30.V.2004, 1 specimen; Kalecik, 40°14′24″N, 33°27′28″E, 842 m, 09.V.2006, 7 specimens; Çankaya, 39°51′57″N, 32°44′25″E, 1050 m, 22.IV.2008, 10 specimens; **Karaman:** Central County, 1410 m, 28.V.2005, 3 specimens; Ermenek, 36°42′18″N, 32°57′18″E, 1483 m, 11.VI.2006, 3 specimens; **Kayseri:** Tomarza, 38°24′27″N, 35°57′05″E, 1370 m, 02.V.2006, 1 specimen; Pınarbaşı, 38°54′11″N, 36°26′49″E, 1579 m, 03.V.2006, 1 specimen; Akkışla, 39°00′59″N, 36°04′16″E, 1235 m, 03.V.2006, 4 specimens; Bünyan, 38°51′24″N, 35°47′11″E, 1233 m, 10.VII.2006, 1 specimen; Sarıoğlan, 39°01′35″N, 36°03′27″E, 1229 m, 10.VII.2006, 1 specimen; **Kırıkkale:** Keskin, 39°38′23″N, 33°38′07″E, 1060 m, 25.VI.2004, 1 specimen; **Kırşehir:** Boztepe, 39°24′20″N, 34°15′45″E, 1163 m, 25.VI.2004, 1 specimen; Kaman, 25.VI.2004, 1 specimen; Aktepe Akpınar-Akçakent yolu, 39°31′12″N, 34°03′27″E, 990 m, 28.IV.2006, 1 specimen; Akpınar, 39°26′54″N, 33°58′58″E, 1157 m, 05.VII.2006, 1 specimen; Akpınar, 39°26′43″N, 34°05′50″E, 1309 m, 05.VII.2006, 1 specimen; **Nevşehir:** HacıBektaş, 38°54′19″N, 34°38′09″E, 1109 m, 23.VI.2007, 3 specimens; **Niğde:** Central County, 38°00′38″N, 34°50′33″E, 1545 m, 23.IV.2006, 1 specimen; **Sivas:** Yıldızeli, 39°49′44″N, 36°42′16″E, 1339 m, 22.VII.2005, 1 specimen; Divriği, 39°30′27″N, 38°07′32″E, 1405 m, 22.V.2006, 5 specimens; Central County, 39°38′28″N, 37°00′29″E, 1338 m, 22.V.2006, 1 specimen; Ulaş, 39°34′14″N, 36°58′03″E, 1389 m, 23.V.2006, 1 specimen; Gemerek, 39°17′12″N, 36°00′25″E, 1187 m, 23.V.2006, 1 specimen; Gemerek, 39°18′18″N, 35°56′48″E, 1345 m, 23.V.2006, 1 specimen; Central County, 39°24′17″N, 36°41′25″E, 1366 m, 23.V.2006, 2 specimens; Yıldızeli, 39°59′49″N, 36°40′17″E, 1450 m, 24.V.2006, 1 specimen; Yıldızeli, 39°49′51″N, 36°42′10″E, 1338 m, 15.VII.2006, 3 specimens; Zara, 39°39′35″N, 37°45′49″E, 1494 m, 01.VI.2007, 4 specimens; **Yozgat:** Sarıkaya, 39°33′31″N, 35°34′00″E, 1145 m, 14.VII.2006, 1 specimen; Central County, 39°38′07″N, 37°55′21″E, 1040 m, 04.V.2007, 1 specimen; Sorgun, 40°04′07″N, 35°15′23″E, 1545 m, 19.V.2008, 1 specimen; col. M. Kabalak. **Ankara:** Haymana, 39°33′41″N, 32°42′55″E, 1037 m, 28.V.2008, 1 specimen; Ayaş, 40°01′43″N, 32°22′39″E, 1087 m, 29.V.2008, 2 specimens; **Karaman:** Başyayla, 36°45′15″N, 32°40′32″E, 1301 m, 13.VI.2009, 1 specimen; **Kayseri:** Felahiye, 39°07′36″N, 35°38′08″E, 1397 m, 21.VII.2009, 1 specimen; **Sivas:** Hafik, 39°49′34″N, 37°16′27″E, 1294 m, 18.VII.2009, 1 specimen; Hafik, 39°55′12″N, 37°23′44″E, 1322 m, 18.VII.2009, 1 specimen; col. Y. Turan.

**Subfamily: MELANOTINAE Candèze, 1859 Genus: *Melanotus* Eschscholtz, 1829 Subgenus: *Melanotus* Eschscholtz, 1829 39. *Melanotus* (s. str.) *bajulus* (Erichson, 1841)**
Material Examined: **Yozgat:** Sorgun, 40°04′08″N, 35°15′21″E, 1536 m, 22.VII.2005, 1 specimen; col. M. Kabalak.

**40. *Melanotus* (s. str.) *fraseri* Platia and Schimmel, 1993**
Material Examined: **Ankara:** Çankaya, 39°54′38″N, 32°49′04″E, 909 m, 31.VII.2002, col. A. Gültekin, 1 specimen. **Ankara:** Çubuk, 40°17′15″N, 33°00′53″E, 1139 m, 17.VII.2005, 1 specimen; Çankaya, 39°52′17″N, 32°44′08″E, 1042 m, 02.VIII.2005, 2 specimens; Çankaya, 39°52′17″N, 32°44′08″E, 1042 m, 29.VII.2008, 2 specimens; col. M. Kabalak.

**41. *Melanotus* (s. str.) *fusciceps* Gyllenhal, 1817**
Material Examined: **Ankara:** Yenimahalle, 39°57′58″N, 32°48′21″E, 885 m, 18.VII.2002, 1 specimen; Ayaş, 40°02′38″N, 32°15′24″E, 728 m, 25.VII.2002, 5 specimens; Bala, 39°39′31″N, 33°04′22″E, 970 m, 26.VII.2002, 1 specimen; Gölbaşı, 39°44′11″N, 32°50′02″E, 1084 m, 01.VIII.2002, 4 specimens; Yenimahalle, 39°57′58″N, 32°48′21″E, 885 m, 16.VIII.2002, 1 specimen; Çankaya, 39°52′17″N, 32°44′08″E, 1042 m, 02.VIII.2005, 1 specimen; Yenimahalle, 39°57′58″N, 32°48′21″E, 885 m, 08.VIII.2002, 1 specimen; Elmadağ, 39°58′23″N, 33°07′06″E, 1110 m, 10.VIII.2006, 1 specimen; Sincan, 39°59′36″N, 32°35′28″E, 835 m, 10.VII.2007, 1 specimen; Bahçelievler, 39°55′16″N, 32°49′35″E, 902 m, 29.VII.2008, 1 specimen; Çankaya, 39°52′17″N, 32°44′08″E, 1042 m, 29.VII.2008, 1 specimen; Elmadağ, 39°58′23″N, 33°07′06″E, 1110 m, 29.VII.2009, 1 specimen; Bahçelievler, 39°55′16″N, 32°49′35″E, 902 m, 06.VIII.2009, 1 specimen; **Eskişehir:** Mihalıççık, 39°58′28″N, 31°22′38″E, 933 m, 24.VII.2002, 1 specimen; **Kayseri:** Central County, 38°53′31″N, 35°44′05″E, 1216 m, 10.VII.2006, 1 specimen; **Nevşehir:** Avanos, 38°58′37″N, 34°57′57″E, 1210 m, 06.VII.2006, 1 specimen; **Sivas:** Central County, 39°41′57″N, 37°0′33″E, 1255 m, 20.VII.2005, 1 specimen; **Yozgat:** Central County, 39°38′07″N, 34°55′25″E, 1027 m, 12.VII.2006, 5 specimens; col. M. Kabalak.

**Subfamily: NEGASTRIINAE  Genus: *Quasimus* Des Gozis, 1886 42. *Quasimus minutissimus* (Germar, 1817)**
Material Examined: **Çankırı:** Ilgaz, 41°4′22″N, 33°43′47″E, 1851 m, 22.VII.2006, 7 specimens; **Sivas:** Koyulhisar, 40°21′21″N, 37°51′17″E, 1644 m, 02.VI.2007, 1 specimen; **Yozgat:** Sorgun, 40°04′08″N, 35°15′21″E, 1536 m, 22.VII.2005, 1 specimen; Sorgun, 39°54′47″N, 35°18′04″E,, 1224 m, 05.V.2006, 60 specimens; col. M. Kabalak.

**Genus: *Zorochros* C. G. Thomson, 1859 43. *Zorochros dermestoides* (Herbst, 1806)**
Material Examined: **Ankara:** Çubuk, 40°26′48″N, 32°52′38.11″E, 1418 m, 29.V.2003, col. M. Kabalak, 2 specimens; **Eskişehir:** Mihalıççık, 39°59′13″N, 31°20′32″E, 643 m, 30.VI.2009, 1 specimen; col. Y. Turan.

**44. *Zorochros georgicus* Dolin and Tschatlandze, 1980**
Material Examined: **Karaman:** Ermenek, 36°42′18″N, 32°57′18″E, 1483 m, 11.VI.2006, 1 specimen; col. M. Kabalak.

**45. *Zorochros heyrovskyi* Roubal, 1940**
Material Examined: **Konya:** Kadınhanı, 38°08′42″N, 32°07′33″E, 1319 m, 17.IV.2006, 15 specimens; Taşkent, 36°50′26″N, 32°29′36″E, 1820 m, 10.VI.2006, 5 specimens; **Yozgat:** Sorgun, 39°54′47″N, 35°18′04″E, 1224 m, 05.V.2006, 6 specimens; col. M. Kabalak.

**46. *Zorochros pilosellus* (Reitter, 1895)**
Material Examined: **Karaman:** Central County, 36°56′49″N, 33°02′20″E, 408 m, 28.V.2005, 4 specimens; col. M. Kabalak.

**47. *Zorochros stibicki* (Leseigneur 1970)**
Material Examined: **Ankara:** Kızılcahamam, 40°38′23″N, 32°30′22″E, 1369 m, 21.V.2003, 1 specimen; col. M. Kabalak.

**Subfamily: Agrypninae Candèze, 1857 Tribe: Agrypnini Candèze, 1857 Genus: *Agrypnus* Eschscholtz, 1829 48. *Agrypnus crenicollis* (Ménétriés, 1832)**
Material Examined: **Sivas:** Suşehri, 40°01′12″N, 38°00′28″E, 1614 m, 14.V.2006, 3 specimens; Suşehri, 40°01′11″N, 38°00′25″E, 1620 m, 02.VI.2007, 4 specimens; col. M. Kabalak.

**Tribe: Oophorini Gistel, 1856 Genus: *Drasterius* Eschscholtz, 1829 49. *Drasterius bimaculatus* Rossi, 1790**
Material Examined: **Ankara:** Etimesgut, 39°59′03″N, 32°37′30″E, 845 m, 10.XI.2001, 3 specimens; col. Y. Güler. **Ankara:** Kızılcahamam, 40°34′51″N, 32°37′21″E, 1163 m, 31.VII.2002, 2 specimens; Ayaş, 39°59′47″N, 32°16′23″E, 747 m, 11.V.2003, 1 specimen; Beypazarı, 40°06′14″N, 32°01′38″E, 522 m, 14.V.2003, 40 specimens; Nallıhan, 40°06′29″N, 31°36′02″E, 467 m, 14.V.2003, 2 specimens; Elmadağ, 39°47′37″N 33°15′25″E 842m 15.V.2003, 20 specimens; Elmadağ 39°53′16″N 33°15′48″E 1038 m, 15.V.2003, 1 specimen; Elmadağ, 39°44′20″N, 33°11′14″E, 898 m, 15.V.2003, 1 specimen; Çubuk, 40°22′55″N, 33°03′50″E, 1277 m, 16.V.2003, 1 specimen; Çubuk, 40°25′47″N, 33°06′44″E, 1545 m, 16.V.2003, 1 specimen; Çubuk, 40°23′47″N, 33°03′50″E, 1261 m, 16.V.2003, 3 specimens; Polatlı, 39°30′12″N, 32°12′57″E, 849 m, 18.V.2003, 6 specimens; Ayaş, 40°05′51″N, 32°15′04″E, 673 m, 24.V.2003, 4 specimens; Güdül, 40°17′20″N, 31°57′24″E, 1398 m, 24.V.2003, 5 specimens; Akyurt, 40°08′40″N, 33°14′59″E, 1171 m, 30.V.2003, 1 specimen; Beypazarı 1.5 km, 40°11′04″N, 31°54′53″E, 716 m, 07.VI.2003, 1 specimen; Beypazarı, 40°19′34″N, 32°03′12″E, 1016 m, 07.VI.2003, 1 specimen; Kızılcahamam, 40°20′00″N, 32°42′03″E, 971 m, 17.VI.2003, 2 specimens; Polatlı, 39°22′29″N, 32°15′57″E, 839 m, 18.VI.2003, 1 specimen; Nallıhan, 40°07′45″N, 31°33′30″E, 620 m, 23.VI.2003, 3 specimens; Nallıhan, 40°13′53″N, 31°20′27″E, 658 m, 23.VI.2003, 15 specimens; Çubuk, 40°18′54″N, 32°55′46″E, 1072 m, 25.VI.2003, 1 specimen; Gölbaşı, 39°29′05″N, 32°49′31″E, 1286 m, 24.VII.2003, 1 specimen; Ayaş, 40°06′09″N, 32°14′58″E, 687 m, 09.VII.2003, 1 specimen; Çubuk, 40°14′19″N, 33°11′05″E, 1132 m, 04.IX.2003, 1 specimen; **Çankırı:** Bayramören, 40°57′01″N, 33°11′57″E, 765 m, 04.VI.2008, 1 specimen; **Karaman:** Central County, 36°56′49″N, 33°02′20″E, 416 m, 28.V.2005, 4 specimens; **Kayseri:** Develi, 38°23′21″N, 35°21′52″E, 1075 m, 12.IV.2003, 9 specimens; Develi, 38°23′21″N, 35°21′52″E, 1075 m, 28.VII.2003, 1 specimen; Develi, 38°23′21″N, 35°21′52″E, 1072 m, 01.V.2006, 8 specimens; **Kırıkkale:** Çelebi, 39°30′36″N, 33°31′06″E, 993 m, 25.VII.2003, 1 specimen; **Konya:** Doğanhisar, 38°09′26″N, 31°39′41″E, 1196 m, 14.V.2005, 8 specimens; Tuzlukçu, 38°26′31″N, 31°39′53″E, 1002 m, 16.IV.2006, 1 specimen; Tuzlukçu, 38°30′13″N, 31°35′44″E, 953 m, 16.IV.2006, 1 specimen; Akşehir, 38°26′31″N, 31°39′53″E, 1002 m, 16.IV.2006, 3 specimens; Kadınhanı, 38°08′42″N, 32°07′33″E, 1319 m, 17.IV.2006, 1 specimen; Ilgın, 38°16′23″N, 31°42′25″E, 1101 m, 17.IV.2006, 1 specimen; Beyşehir, 37°38′27″N, 31°37′44″E, 1128 m, 18.IV.2006, 1 specimen; Akşehir, 38°26′57″N, 31°31′02″E 967 m, 03.V.2007, 2 specimens; Beyşehir, 37°37′22″N, 31°27′09″E, 1126 m, 04.V.2007, 9 specimens; Bozkır, 37°06′50″N, 32°18′17″E, 1277 m, 05.V.2007, 2 specimens; **Niğde:** Çamardı, 37°52′43″N, 35°06′25″E, 1565 m, 21.VI.2006, 1 specimen; Sivas, 40°14′40″N, 38°05′09″E, 752 m, 02.VI.2007, 1 specimen; **Yozgat:** Çekerek, 40°04′11″N, 35°33′46″E, 788 m, 12.VI.2007, 1 specimen; col. M. Kabalak. **Aksaray:** Central County, 38°16′42″N, 33°42′20″E, 926 m, 29.V.2004, 1 specimen; Şereflikoçhisar, 38°56′20″N, 33°32′18″E, 948 m, 29.V.2004, 2 specimens; **Kırşehir:** Boztepe, 39°24′20″N, 34°15′45″E, 1163 m, 25.VI.2004, 2 specimens; col. N. Kocatepe. **Ankara:** Çankaya, 39°52′00″N, 32°44′27″E, 1046 m, 22.V.2007, 1 specimen; Çankaya, 39°53′00″N, 32°45′21″E, 936 m, 12.IV.2008, 1 specimen; col. K. Koyuncu. **Ankara:** Yenimahalle, 39°53′22″N, 32°42′02″E, 932 m, 07.IX.2008, col. O. Sert, 1 specimen. **Aksaray:** Güzelyurt, 38°15′15″N, 34°18′10″E, 1227 m, 15.VI.2009, 2 specimens; Güzelyurt, 38°16′03″N, 34°17′16″E, 1196 m, 22.VI.2009, 6 specimens; Gülağaç, 38°21′19″N, 34°13′45″E, 1107 m, 23.VI.2009, 3 specimens; **Ankara:** Şereflikoçhisar, 38°45′48″N, 33°41′54″E, 1094 m, 27.V.2008, 1 specimen; Şereflikoçhisar, 38°51′59″N, 33°42′21″E, 1064 m, 27.V.2008, 1 specimen; Şereflikoçhisar, 38°55′04″N, 33°39′21″E, 1034 m, 27.V.2008, 1 specimen; Çubuk, 40°23′19″N, 32°56′45″E, 1183 m, 13.VI.2008, 1 specimen; Ayaş, 39°59′45″N, 32°16′26″E, 804 m, 14.VI.2008, 1 specimen; Nallıhan, 40°04′17″N, 31°23′44″E, 412 m, 14.VI.2008, 2 specimens; Beypazarı, 40°20′20″N, 31°55′50″E, 1632 m, 15.VI.2008, 1 specimen; **Eskişehir:** Sivrihisar, 39°40′43″N, 31°46′18″E, 960 m, 30.VI.2009, 1 specimen; Mihalıççık, 39°59′13″N, 31°20′32″E 643 m, 30.VI.2009, 1 specimen; Central County, 39°57′16″N, 30°30′10″E, 1026 m, 01.VII.2009, 1 specimen; Sivrihisar, 39°11′25″N, 31°37′39″E, 838 m, 01.VII.2009, 1 specimen; **Kayseri:** Yahyalı, 37°53′49″N, 35°30′49″E, 800 m, 22.VII.2009, 1 specimen; **Karaman:** Ayrancı, 37°10′47″N, 33°47′22″E, 1444 m, 14.VI.2009, 2 specimens; **Kırşehir:** Akpınar, 39°31′05″N, 33°51′43″E, 938 m, 28.VI.2009, 1 specimen; **Konya:** Central County, 37°53′19″N 32°18′35″E 1257m 1 specimen 02.VI.2009, Central County 37°47′53″N 32°10′39″E, 1405 m, 02.VI.2009, 1 specimen; Seydişehir, 37°32′50″N, 32°09′15″E, 1610 m, 02.VI.2009, 1 specimen; Hadim, 37°03′01″N, 32°03′33″E, 1283 m, 04.VI.2009, 2 specimens; Ereğli, 37°25′45″N, 34°11′36″E, 1160 m, 14.VI.2009, 5 specimens; **Nevşehir:** Avanos, 38°44′56″N, 34°54′06″E, 1026 m, 24.VI.2009, 1 specimen; Avanos, 38°43′59″N, 34°55′50″E, 938 m, 24.VI.2009, 1 specimen; Ürgüp, 38°38′59″N, 35°00′36″E, 1332 m, 24.VI.2009, 1 specimen; Gülşehir, 38°42′48″N, 34°29′04″E, 972 m, 25.VI.2009, 3 specimens; Avanos, 39°13′17″N, 34°51′09″E, 1045 m, 26.VI.2009, 1 specimen; **Sivas:** Doğanşar, 40°15′19″N, 37°32′45″E, 1069 m, 18.VII.2009, 3 specimens; Suşehri, 40°01′21″N, 38°10′51″E, 1155 m, 19.VII.2009, 1 specimen; Ulaş, 39°25′21″N, 36°52′26″E, 1458 m, 20.VII.2009, 1 specimen; col. Y Turan.

**Genus: *Aeoloderma* Fleutiaux, 1928 50. *Aeoloderma crucifer* (Rossi, 1790)**
Material Examined: **Ankara:** Polatlı, 39°30′12″N, 32°12′56″E, 848 m, 18.VI.2003, 3 specimens; **Konya:** Akşehir, 38°26′57″N, 31°31′02″E, 967 m, 03.V.2007, 1 specimen; **Nevşehir:** Hacıbektaş, 38°45′49″N, 34°35′43″E, 949,m, 29.IV.2006, 1 specimen; col. M. Kabalak

**Genus: *Aeolosomus* Dolin, 1982 51. *Aeolosomus rossi* (Germar, 1844)**
Material Examined: **Konya:** Akşehir, 38°26′50″N, 31°30′58″E, 966 m, 13.V.2005, 6 specimens; col. M. Kabalak.

**Subfamily: Cardiophorinae Candèze, 1860 Tribe: Cardiophorini Candèze, 1859 Genus: *Cardiophorus* Eschscholtz, 1829 Subgenus: *Cardiophorus* Eschscholtz, 1829 52. *Cardiophorus* (*Cardiophorus*) *analis* Schwarz, 1892**
Material Examined: **Konya:** Beyşehir, 37°43′59″N, 31°41′18″E, 1127 m, 28.V.1999, 4 specimens; col. O. Sert.

**53. *Cardiophorus* (s. str.) *anticus* Erichson, 1840**
Material Examined: **Ankara:** Kızılcahamam,40°20′00″N, 32°42′02″E, 971 m, 17.VI.2003, 1 specimen; Çankaya, 39°51′57″N, 32°44′25″E, 1049 m, 05.V.2005, 1 specimen; Çankaya, 39°51′57″N, 32°44′25″E, 1049 m, 02.V.2008, 2 specimens; col. M. Kabalak.

**54. *Cardiophorus* (s. str.) *discicollis* (Herbst, 1806)**
Material Examined: **Eskişehir:** Sivrihisar, 39°32′35″N, 31°42′39″E, 815 m, 06.VI.2006, 1 specimen; **Karaman:** Central County, 37°08′27″N, 33°34′09″E, 1260 m, 07.V.2007, 3 specimens; col. M. Kabalak. **Aksaray:** Güzelyurt, 38°15′13″N, 34°18′07″E, 1266 m, 15.V.2009, 1 specimen; **Karaman:** Ayrancı, 37°21′33″N, 33°38′52″E, 1112 m, 14.VI.2009, 1 specimen; col. Y. Turan.

**55. *Cardiophorus* (s. str.) *dolini* Mardjanian, 1985**
Material Examined: **Ankara:** Gölbaşı, 39°45′44″N, 32°45′29″E, 987 m, 20.V.2003, 5 specimens; Güdül, 40°12′35″N, 32°14′15″E, 703 m, 24.V.2003, 3 specimens; Haymana, 39°26′03″N, 32°27′10″E, 1198 m, 25.V.2003, 3 specimens; Kazan, 40°07′37″N, 32°35′36″E, 856 m, 16.VI.2003, 1 specimen; Bala, 39°37′54″N, 32°55′08″E, 1174 m, 21.VI.2003, 1 specimen; Bala, 39°30′55″N, 32°57′32″E, 1044 m, 21.VI.2003, 2 specimens; Gölbaşı, 39°29′05″N, 32°49′31″E, 1287 m, 24.VI.2003, 13 specimens; Gölbaşı, 39°35′56″N, 32°50′45″E, 1096 m, 24.VI.2003, 2 specimens; Gölbaşı, 24.VI.2003; **Eskişehir:** Seyitgazi, 39°28′50″N, 30°39′42″E, 1000 m, 05.VI.2006, 2 specimens; **Karaman:** Merkez, 37°26′18″N, 33°10′00″E, 1210 m, 21.IV.2006, 1 specimen; **Kayseri:** Tomarza, 38°30′13″N, 35°48′02″E, 1442 m, 29.V.2007, 3 specimens; Sarıoğlan, 39°04′13″N, 35°50′37″E, 1177 m, 30.V.2007, 1 specimen; **Kırşehir:** Kaman, 39°22′13″N, 33°50′20″E, 1118 m, 25.VI.2004, 1 specimen; Central County, 39°21′49″N, 34°10′55″E, 1208 m, 09.VI.2007, 2 specimens; Çiçekdağı, 39°34′16″N, 34°27′13″E, 904 m, 10.VI.2007, 1 specimen; Mucur, 39°07′14″N, 34°25′06″E, 1189 m, 10.VI.2007, 8 specimens; Mucur, 39°01′21″N, 34°16′45″E, 975 m, 10.VI.2007, 12 specimens; **Konya:** Kulu, 39°03′47″N, 32°53′52″E, 1070 m, 13.V.2005, 25 specimens; Central County, 37°40′59″N, 32°35′11″E, 1015 m, 27.V.2005, 1 specimen; Kulu, 39°03′46″N, 32°53′46″E, 1068 m, 09.VI.2006, 4 specimens; **Nevşehir:** Kozaklı, 39°19′03″N, 34°44′49″E, 1008 m, 10.VII.2007, 18 specimens; **Yozgat:** Boğazlıyan, 39°22′53″N, 35°08′08″E, 1177 m, 11.VI.2007, 7 specimens; Boğazlıyan, 39°15′30″N, 35°12′35″E, 1148 m, 11.VI.2007, 11 specimens; col. M. Kabalak. **Ankara:** Şereflikoçhisar, 39°05′19″N, 33°32′06″E, 947 m, 25.V.2008, 14 specimens; Beypazarı, 40°16′49″N, 31°55′31″E, 1136 m, 17.VI.2008, 1 specimen; **Nevşehir:** Gülşehir, 38°45′56″N, 34°35′59″E, 909 m, 26.VI.2009, 8 specimens; col. Y. Turan.

**56. *Cardiophorus* (s. str.) *frequens* Platia and Gudenzi, 2002**
Material Examined: **Konya:** Karapınar, 37°42′49″N, 33°31′34″E, 1001 m, 20.IV.2006, 3 specimens; Bozkır, 37°06′50″N, 32°18′17″E, 1277 m, 05.V.2007, 1 specimen; col. M. Kabalak.

**57. *Cardiophorus* (s. str.) *impressiventris* Schwarz, 1900**
Material Examined: **Karaman:** Central County, 37°26′18″N, 33°10′00″E, 1210 m, 21.IV.2006, 8 specimens; col. M. Kabalak.

**58. *Cardiophorus* (s. str.) *kindermanni* Candèze, 1860**
Material Examined: **Karaman:** Central County, 36°56′49″N, 33 02′20″E, 416 m, 11.VI.2006, 3 specimens; Ermenek, 36°42′18″N, 32°57′18″E, 1483 m, 11.VI.2006, 1 specimen; Central County, 37°07′13″N, 33°04′21″E, 1156 m, 06.V.2007, 1 specimen; col. M. Kabalak.

**59. *Cardiophorus* (s. str.) *levis* Platia and Gudenzi, 2002**
Material Examined: **Yozgat:** Sorgun, 40º04′27″N, 35º14′41″E, 1442 m, 19.V.2008, 1 specimen; col. M. Kabalak.

**60. *Cardiophorus* (s. str.) *megathorax* Faldermann, 1835**
Material Examined: **Ankara:** Polatlı, 39°25′15″N, 32°15′01″E, 916 m, 18.VI.2003, 8 specimens; Bala, 39°30′02″N, 33°16′56″E, 951 m, 21.VI.2003, 1 specimen; Şereflikoçhisar, 39°10′08″N, 33°22′38″E, 1029 m, 21.VI.2003, 4 specimens; Çubuk, 40°12′35″N, 32°53′26″E, 1317 m, 25.VI.2003, 2 specimens; **Kayseri:** İncesu, 38°40′33″N, 35°13′48″E, 1056 m, 27.V.2007, 3 specimens; Sarıoğlan, 39°04′13″N, 35°50′37″E, 1177 m, 30.V.2007, 1 specimen; İncesu, 38°41′33″N, 35°07′41″E, 1222 m, 24.VI.2007, 16 specimens; **Konya:** Kulu, 39°08′39″N, 33°06′29″E, 1005 m, 09.VI.2006, 1 specimen; Kulu, 39°03′46″N, 32°53′46″E, 1068 m, 09.VI.2006, 20 specimens; **Nevşehir**: Central County, 38°36′28″N, 34°48′35″E, 1487 m, 25.VI.2007, 6 specimens; Acıgöl, 38°30′05″N, 34°29′52″E, 1311 m, 25.VI.2007, 1 specimen; **Yozgat**: Çekerek, 40°04′11″N, 35°33′46″E, 788 m, 12.VI.2007, 1 specimen; col. M. Kabalak. **Aksaray**: Central County, 38°11′37″N, 34°07′15″E, 1287 m, 15.VI.2009, 4 specimens; **Ankara**: Şereflikoçhisar, 39°00′00″′N, 33°30′31″E, 1002 m, 25.V.2008, 1 specimen; **Eskişehir**: Mihalıççık 39°45′32″N, 31°34′28″E 764 m, 30.VI.2009, 1 specimen; **Karaman**: Central County, 37°13′28″N, 33°02′10″E, 1040 m, 12.VI.2009, 11 specimens; Ayrancı, 37°21′33″N, 33°38′52″E, 1112 m, 14.VI.2009, 6 specimens; **Konya**: Hadim, 37°01′35″N, 32°42′08″E, 814 m, 04.VI.2009, 1 specimen; col. Y. Turan.

**61. *Cardiophorus* (s. str.) *miniaticollis* Candèze, 1860**
Material Examined: **Konya:** Güneysınır, 37°17′54″N, 32°43′05″E, 1130 m, 19.IV.2006, specimen; col. M. Kabalak.

**62. *Cardiophorus* (s. str.) *nigratissimus* Buysson, 1891**
Material Examined: **Ankara:** Polatlı, 39°25′15″N, 32°15′01″E, 916 m, 18.VI.2003, 32 specimens; Şereflikoçhisar, 39°10′08″N, 33°22′38″E, 1029 m, 21.VI.2003, 17 specimens; Nallıhan, 40°07′45″N, 31°32′38″E, 633 m, 23.VI.2003, 3 specimens; Çubuk, 40°12′35″N, 32°53′26″E, 1317 m, 25.VI.2003, 7 specimens; **Kayseri:** İncesu, 38°41′33″N, 35°07′41″E, 1222 m, 24.VI.2007, 51 specimens; İncesu, 38°40′33″N, 35°13′48″E, 1056 m, 27.V.2007, 3 specimens; **Kırşehir:** Çiçekdağı, 39°34′16″N, 34°27′13″E, 904 m, 10.VI.2007, 1 specimen; Mucur, 38°52′57″N, 34°20′20″E, 906 m, 23.VI.2007, 1 specimen; **Konya:** Kulu, 39°03′46″N, 32°53′46″E, 1068 m, 09.VI.2006, 37 specimens; **Nevşehir:** Gülşehir, 38°48′21″N, 34°30′56″E, 928 m, 26.VI.2004, 2 specimens; Hacıbektaş, 38°51′01″N, 34°33′33″E, 1255 m, 23.VI.2007, 34 specimens; Gülşehir, 38°44′29″N, 34°25′19″E, 1005 m, 23.VI.2007, 1 specimen; Derinkuyu, 38°21′10″N, 34°37′29″E, 1304 m, 25.VI.2007, 2 specimens; Acıgöl, 38°30′05″N, 34°29′52E, 1311 m, 25.VI.2007, 2 specimens; Kozaklı, 39°06′39″N, 34°50′16″E, 1167 m, 25.VI.2007, 42 specimens; **Sivas:** Divriği, 39°17′26″N, 38°01′43″E, 1185 m, 01.VI.2007, 29 specimens; **Yozgat:** Çekerek, 40°04′11″N, 35°33′46″E, 788 m, 12.VI.2007, 1 specimen; Aydıncık, 40°10′31″N, 35°21′33″E, 684 m, 12.VI.2007, 1 specimen; col. M. Kabalak. **Aksaray:** Eskil, 38°16′03″N, 34°17′16″E, 964 m, 22.VI.2009, 1 specimen; **Eskişehir:** Alpu, 39°40′48″N, 31°03′44″E, 811 m, 03.VII.2009, 1 specimen; **Karaman:** Central County, 37°13′28″N, 33°02′10″E, 1040 m, 12.VI.2009, 12 specimens; Ayrancı, 37°21′33″N, 33°38′52″E, 1112 m, 14.VI.2009, 4 specimens; col. Y. Turan.

**63. *Cardiophorus* (s. str.) *parvulus* Platia and Gudenzi, 2000**
Material Examined: **Karaman:** Central County, 36°56′37″N, 33°16′28″E, 1465 m, 28.V.2005, 2 specimens; Central County, 36°56′49″N, 33°2′20″E, 416 m, 28.V.2005, 3 specimens; **Konya:** Hadim, 37°06′28″N, 32°27′20″E, 1229 m, 27.V.2005, 1 specimen; Cihanbeyli, 38°48′44″N, 32°54′03″E, 963 m, 03.V.2007, 1 specimen; col. M. Kabalak. **Aksaray:** Güzelyurt, 38°15′13″N, 34°18′07″E, 1266 m, 15.VI.2009, 5 specimens; col. Y. Turan.

**64. *Cardiophorus* (s. str.) *ruficruris* (Brullé, 1832)**
Material Examined: **Ankara:** Ayaş, 39°52′58″N, 32°35′31″E, 682 m, 11.V.2003, 3 specimens; **Konya:** Cihanbeyli, 38°48′44″N, 32°54′03″E, 963 m, 03.V.2007, 1 specimen; col. M. Kabalak.

**65. *Cardiophorus* (s. str.) *sacratus* Erichson, 1840**
Material Examined: **Konya:** Yunak, 38°55′03″N, 32°05′25″E, 926 m, 03.V.2007, 1 specimen; Hadim, 37°00′44″N, 32°19′52″E, 1380 m, 05.V.2007, 1 specimen; **Nevşehir:** Hacıbektaş, 39°00′24″N, 34°43′44″E, 1184 m, 29.IV.2006, 1 specimen; **Sivas:** Koyulhisar, 40°16′46″N, 37°49′35″E, 671 m, 14.V.2006, 1 specimen; Gemerek, 39°18′18″E, 35°56′48″E, 1345 m, 23.V.2006, 1 specimen; **Yozgat:** Aydıncık, 40°13′12″N, 35°18′56″E, 639 m, 06.V.2006, 1 specimen; Aydıncık, 40°13′12″N, 35°18′56″E, 639 m, 22.V.2007, 1 specimen; Akdağmadeni, 39°31′30″N, 36°00′40″E, 1921 m, 10.VII.2007, 1 specimen; col. M. Kabalak.

**66. *Cardiophorus* (s. str.) *vestigialis* Erichson, 1840**
Material Examined: **Eskişehir:** Sivrihisar, 39°22′55″N, 31°29′28″E, 916 m, 14.V.2007, 1 specimen; **Kayseri:** Yeşilhisar, 38°21′34″N, 34°58′53″E, 1324 m, 07.VII.2006, 1 specimen; **Konya:** Karapınar, 37°42′48″N, 33°31′23″E, 996 m, 20.IV.2006, 5 specimens; col. M. Kabalak.

**Genus: *Dicronychus* Brullè, 1832 67. *Dicronychus adanensis* (Pic, 1908)**
Material Examined: **Yozgat:** Aydıncık, 40°13′12″N, 35°18′56″E, 639 m, 06.V.2006, 8 specimens; col. M. Kabalak.

**68. *Dicronychus cinereus* (Herbst, 1784)**
Material Examined: **Çankırı:** Central County, 40°38′05″N, 33°36′28″E, 754 m, 05.VI.2008, 2 specimens; **Eskişehir:** Mihalıççık, 39°59′12″N, 31°20′33″E, 646 m, 04.VI.2006, 1 specimen; **Karaman:** Central County, 37°08′27″N, 33°34′09″E, 1260 m, 07.V.2007, 1 specimen; **Kayseri:** Yeşilhisar, 38°20′39″N, 34°58′17″E, 1315 m, 27.V.2007, 2 specimens; Yeşilhisar, 38°21′34″N, 34°58′53″E, 1324 m, 27.V.2007, 1 specimen; **Niğde:** Çamardı, 37°51′31″N, 34°57′30″E 1580 m, 29.V.2005, 47 specimens; Çamardı, 37°48′40″N, 34°59′40″E, 1425 m, 21.VI.2006, 1 specimen; **Sivas:** Gemerek, 39°19′15″N, 35°51′44″E, 1470 m, 23.V.2006, 4 specimens; **Yozgat:** Çekerek, 40°5′13″N, 35°35′14″E, 775 m, 06.V.2006, 8 specimens; col. M. Kabalak.

**69. *Dicronychus iconiensis* Pic, 1908**
Material Examined: **Karaman:** Kılbasan, 37°26′18″N, 33°10′00″E, 1210 m, 21.IV.2006, 3 specimens; Kılbasan, 37°25′40″N, 33°07′03″E, 1534 m, 21.IV.2006, 1 specimen; **Kayseri:** Develi, 38°27′59″N, 35°31′05″E, 1834 m, 28.V.2007, 2 specimens; **Konya:** Obruk, 38°10′27″N, 33°10′58″E, 994 m, 15.IV.2006, 2 specimens; Akşehir, 38°30′14″N, 31°35′43″E, 953 m, 16.IV.2006, 4 specimens; Akşehir, 38°26′31″N, 31°39′53″E, 1002 m, 16.IV.2006, 1 specimen; Seydişehir, 37°23′33″N, 31°59′26″E, 1096 m, 18.IV.2006, 1 specimen; Cihanbeyli, 38°48′49″N, 32°54′03″E, 963 m, 03.V.2007, 9 specimens; **Niğde:** Çamardı, 37°51′33″N, 34°57′30″E, 1585 m, 23.IV.2006, 1 specimen; **Yozgat:** Aydıncık, 40°13′12″N, 35°18′56″E, 639 m, 06.V.2006, 1 specimen; col. M. Kabalak.

**70. *Dicronychus obscuripennis* (Pic, 1899)**
Material Examined: **Konya:** Cihanbeyli, 38°40′30″N, 33°07′13″E, 927 m, 15.IV.2006, 1 specimen; col. M. Kabalak.

**71. *Dicronychus senaci* Desbrochers des Loges, 1870**
Material Examined: **Konya:** Cihanbeyli, 38°40′30″N, 33°7′13″E, 927 m, 15.IV.2006, 2 specimens; **Sivas:** Gemerek, 39°28′40″N, 35°57′59″E, 1809 m, 03.VI.2007, 3 specimens; col. M. Kabalak.

**Subfamily: Lissominae Laporte, 1835 Genus: *Drapetes* Dejean, 1821 72. *Drapetes mordelloides* (Host, 1789).**
Material Examined: **Kırşehir:** Akpınar, 39°26′43″N, 34°05′50″E, 1309 m, 05.VII.2006, 1 specimen; **Nevşehir:** Ürgüp, 38°36′04″N, 34°54′24″E, 1092 m, 07.VII.2006, 1 specimen; **Sivas:** Şarkışla, 39°34′27″N, 36°14′36″E, 1351 m, 03.VI.2007, 1 specimen; col. M. Kabalak.

### Faunistic composition of the Central Anatolian Region

As a result of this study, it was found that the major part of the Elateridae fauna of the Central Anatolian region is formed by the subfamilies Elaterinae (27 species; 37.5%) and Cardiophorinae (20 species; 27.8%). These subfamilies are followed by the subfamilies Dendrometrinae (11 species; 15.3%), Negastriinae (6 species; 8.3%), Agrypninae (4 species; 5.5%), Melanotinae (3 species; 4.2%) and Lissominae (1 species; 1.4%) (Figure 3) in species richness. The genus ***Cardiophorus*** is the richest genus with 15 species from the Central Anatolian region (Figure 4). The genus ***Cardiophorus*** is followed by ***Agriotes*** (9 species), ***Ampedus*** (8 species), ***Dicronychus*** and ***Zorochros*** (5 species each), ***Melanotus*** and ***Peripontius*** (3 species each), ***Athous***, ***Hemicrepidius***, ***Prosternon***, ***Selatosomus*** (2 species each) and ***Aeoloderma***, ***Aeolosomus***, ***Agrypnus***, ***Dalopius***, ***Drapetes***, ***Drasterius***, ***Idotarmonides***, ***Limonius***, ***Limoniscus***, ***Mulsanteus, Nothodes, Quasimus* and *Synaptus*** (1 species each).

The numbers of species in genera found in the Central Anatolian region were compared with the known numbers of species in genera of Turkey according to Mertlik and Platia (2008) (Table 3). It is apparent that the distributions of species in each genus show that Elateridae fauna found in this study are partially consistent with Elateridae fauna of Turkey.

### Ecological properties of fauna

Results show that ***Agriotes paludum*** (629 specimens) is the most abundant species. ***Synaptus filiformis*** (430 specimens), ***Cardiophorus nigratissimus*** (284 specimens), ***Adrastus anatolicus*** (273 specimens), ***Drasterius bimaculatus*** (231 specimens), ***Agriotes sputator*** (155 specimens) and ***Cardiophorus dolini*** (150 specimens) are also abundant species. ***Adrastus montanus, Agriotes propleuralis, Ampedus nigroflavus, Ampedus ochropterus, Cardiophorus levis, Cardiophorus miniaticollis, Dicronychus obscuripennis, Hemicrepidius nigritulus, Idotarmonides anatolicus, Limoniscus elegans, Melanotus bajulus, Prosternon tessellatum, Zorochros georgicus*** and ***Zorochros stibicki*,** are the rarest species, which are represented with one specimen each (Table 1). In the light of these data, it can be stated that species with most collected specimens may have dense populations and species with fewer collected specimens may have sparse populations in nature. However, other factors may have affected the number of specimens collected, including the coincidence of collecting dates with low or high density of populations, effects of collecting habitats on population density and different collecting methods.

Specimens that were collected from different altitudes and vertical distributions of species exhibited differences. Evaluations of vertical distributions of species were made in terms of vertical intervals, which are appointed as 250 meters (A: 0–250 m, B: 251–500 m, C: 501–750 m, D: 751–1000 m, E: 1001–1250 m and F: 1251–1500 m, G: 1501–1750 m, H: 1751–2000 m and I: 2000 m and above). Evaluation of the results showed that there are differences in vertical distributions of species. Accordingly, most of the species were collected from interval E with 43 species. This interval is followed by interval F (38 species), interval D (33 species), interval G (24 species), interval C (16 species), interval H (12 species), interval B (6 species), interval I (2 species) and interval A (1 species) (Figure 5, Table 1). Vertical distributions of species are given in Table 1. ***Agriotes paludum*,** ***Drasterius bimaculatus*** and ***Synaptus filiformis*** were the most diverse species vertically.

**Table 3.**
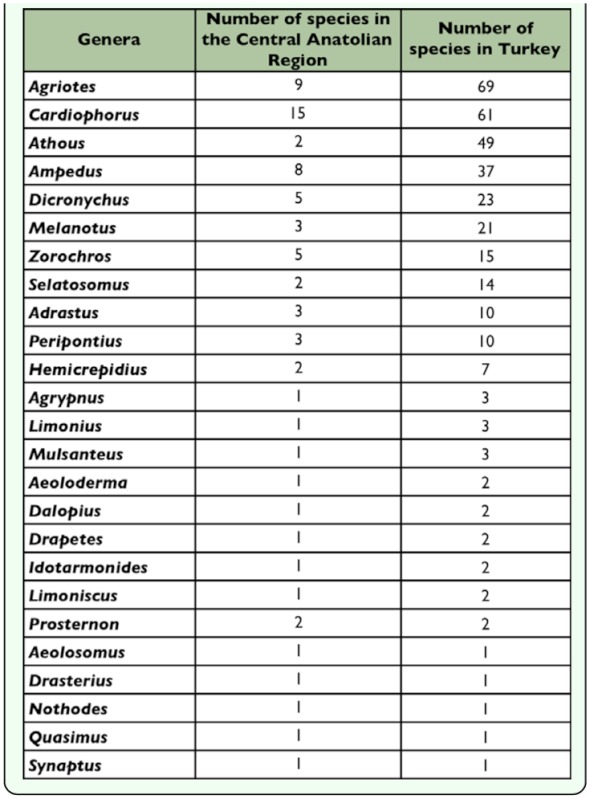
Comparison of the number of species in genera in the Elateridae between the Central Anatolian region and Turkey.

During the fieldwork, species were collected from different habitats using different collecting methods. Most of the species were collected from herbaceous plants near streams (25 species). Other habitats included herbaceous plants near fields and roads (23 species), under stones and plants (12 species), under stones and debris near streams (10 species), forest ground herbaceous plants (10 species), decaying trees (*Populus* spp. and *Salix* spp.) (eight species), trees and bushes (seven species), light trap (six species). ***Cardiophorus miniaticollis*** was collected incidentally inside buildings. Evaluating the collection methods showed that 47 species were collected using insect nets, 30 species using an aspirator, 7 species using the Japanese umbrella, and 6 species using light traps (Table 1). Adults of most detected species occur on herbaceous plants (near streams, fields, roads and forested ground cover). Most of the rest occur under stones and plants, in decaying trees, under stones and debris near streams. Minor numbers of detected species occur on trees and bushes and nocturnal species, which were collected by using light traps.

Specimens were collected between April – November (21 species in April, 47 in May, 41 in June, 27 in July, 4 in August and 1 in September and November) (Figure 6). It is apparent from these data that species of Elateridae are mainly active in May and June, and that April and July are secondarily appropriate months for collecting Elateridae. The species showed minimum activity in August, September, October and November. Collecting periods of species exhibit differences: four species collected only in April (5.6%), 12 species in May (16.7%), six species in June (8.3%), seven species in July (9.7%), three species in April and May (4.2%), one species in April and June (1.4%), five species in April, May and June (6.9%), five species in April, May, June and July (6.9%), two species in April, May and July (2.8%), 14 species in May and June (19.4%), five species in May, June and July (6.9%); two species in June and July (2.8%), one species in June, July and August (1.4%), three species in July and August (4.2%) and 1 species in April, May, June, July, September and November (1.4%) (Figure 5). Numbers of species varied by month: 29 species were collected for one month, 24 species were collected for two months, eight species were collected for three months, five species were collected for four months and one species **(*Drasterius bimaculatus***) was collected for six months. These data help to estimate the occurrence of the species in nature. ***Drasterius bimaculatus*** occurs for the longest time in nature followed by ***Agriotes paludum, Agriotes proximus, Agriotes sputator, Cardiophorus dolini*** and ***Synaptus filiformis.*** As a result of evaluations of active periods of genera, which are represented more than one species, species of the genus ***Adrastus*** were collected during May–July; species of the genus ***Agriotes*** were collected during AprilJuly; species of the genus ***Ampedus*** were collected during April–June; species of the genus ***Athous*** were collected during April–July; species of the genus ***Cardiophorus*** were collected during April–July; species of the genus ***Dicronychus*** were collected during April–June; species of the genus ***Hemicrepidius*** were collected during June–July; species of the genus ***Melanotus*** were collected during July–August; species of the genus ***Peripontius*** were collected during April–July; species of the genus ***Prosternon*** were collected during June–August; species of the genus ***Selatosomus*** were collected during May–June; species of the genus ***Zorochros*** were collected during April–May. These data may help understanding phenologies of these genera (Table 1).

### Zoogeographical composition of fauna

As a result of the evaluations of distributions of detected species in zoogeographical regions and subregions (Figure 7, Table 2), it is evident that 11 species are endemic to Turkey. The remaining 61 species are shared differently with the European part of the Western Palaearctic (53 species), the Middle East (46 species), Middle Asia (25 species), Siberia (12 species), North Africa (nine species), the Far East (seven species), Nearctic region (two species) and the Australian region (one species). This composition shows that the geographical situation of Turkey, at the intersection of Asia, Africa and Europe, affects its fauna. Research area shares most species with the European part of the Western Palaearctic and, subsequently, Asia (Middle Asia, the Middle East, Siberia and the Far East). Because most of Turkey is a part of Asia, that situation may be evaluated as contradictory. However, Cate (2007) reported that the Elateridae fauna of Turkey share the most species with the European part of the Western Palaearctic region (223 species): Asia (194 species), followed by North Africa (29 species), Nearctic region (6 species), Afrotropical region (one species), the Australian region (one species) and Neotropical region (one species). Cate's (2007) data are consistent with our findings. According to Cate (2007), ***Agriotes lineatus*** and ***A. sputator*** are the most widely distributed species within the research area. ***Aeoloderma crucifer, Ampedus sanguinolentas, Cardiophorus (s.str.) vestigialis, Drapetes mordelloides, Drasterius bimaculatus*** and ***Selatosomus (s.str.) latus*** are also widely distributed species within the research area.

According to the present literature, the distribution of the collected species in Turkey are given in Table 2. Thirty-four species out of 72 are reported for the first time from the Central Anatolian region. Detailed locality records of ***Agriotes modestus, Ampedus nigroflavus, A. pomonae, Dalopius marginatus, Drapetes mordelloides, Idotarmonides anatolicus, Zorochros georgicus, Z. heyrovskyi*** and ***Z. pilosellus*** are given for the first time from the research area and Turkey. The research area shares many species with the Mediterranean (35 species), Eastern Anatolian region (33 species), Black Sea region (32 species), Aegean region (29 species), Marmara region (19 species) and the Southeastern Anatolian region (11 species) (Figure 8). Various researchers have previously recorded 38 species from the studied area. During this research, field studies were done following the political borders of provinces, and not the geographical border of the Central Anatolian region. The borders of geographical regions of Turkey were determined according to geographical, floristic and climatic features, but some parts of the provinces of the research area are situated in other geographical regions. As a result, species sharing data of the Central Anatolian and other geographical regions may be affected by that situation.
